# Reconstituted TAD-size chromatin fibers feature heterogeneous nucleosome clusters

**DOI:** 10.1038/s41598-022-19471-3

**Published:** 2022-09-16

**Authors:** Nikolay Korolev, Anatoly Zinchenko, Aghil Soman, Qinming Chen, Sook Yi Wong, Nikolay V. Berezhnoy, Rajib Basak, Johan R. C. van der Maarel, John van Noort, Lars Nordenskiöld

**Affiliations:** 1 School of Biological Sciences, Nanyang Technological University, 60 Nanyang Drive, Singapore, 637551 Singapore; 2 Graduate School of Environmental Studies, Nagoya University, Furo-Cho, Chikusa-Ku, Nagoya, 464-8601 Japan; 3 Department of Physics, National University of Singapore, Singapore, 117542 Singapore; 4 Huygens-Kamerlingh Ohnes Laboratory, Leiden University, Niels Bohrweg 2, 2333 CA Leiden, The Netherlands; 5 Present Address: Department of Emerging Infectious Diseases, Duke-NUS Medical School, Singapore, 169857 Singapore

**Keywords:** Single-molecule biophysics, Biophysics, Biochemistry, DNA

## Abstract

Large topologically associated domains (TADs) contain irregularly spaced nucleosome clutches, and interactions between such clutches are thought to aid the compaction of these domains. Here, we reconstituted TAD-sized chromatin fibers containing hundreds of nucleosomes on native source human and lambda-phage DNA and compared their mechanical properties at the single-molecule level with shorter ‘601’ arrays with various nucleosome repeat lengths. Fluorescent imaging showed increased compaction upon saturation of the DNA with histones and increasing magnesium concentration. Nucleosome clusters and their structural fluctuations were visualized in confined nanochannels. Force spectroscopy revealed not only similar mechanical properties of the TAD-sized fibers as shorter fibers but also large rupture events, consistent with breaking the interactions between distant clutches of nucleosomes. Though the arrays of native human DNA, lambda-phage and ‘601’ DNA featured minor differences in reconstitution yield and nucleosome stability, the fibers’ global structural and mechanical properties were similar, including the interactions between nucleosome clutches. These single-molecule experiments quantify the mechanical forces that stabilize large TAD-sized chromatin domains consisting of disordered, dynamically interacting nucleosome clutches and their effect on the condensation of large chromatin domains.

## Introduction

Chromatin consists of DNA wrapped in two turns around histone octamers, consisting of two copies of H2A, H2B, H3, and H4 histones. The resulting nucleosomes are connected by linker DNA of variable length, generally below 100 bp. The positioning of nucleosomes is dynamic and varies from cell to cell. Nucleosomes can be repositioned depending on the transcription activity of the underlying genes, with silent chromatin having longer linker DNA^1,2^. Centromeric and telomeric chromatin display short linker DNA, and while certain nucleosomes are well mapped, particularly close to transcription start sites (TSS), other nucleosomes away from the TSS show more positioning freedom. Regions of DNA without nucleosomes, called nucleosome-free regions (NFR), of 100–300 bp length are prevalently found in vivo^2–4^. Overall, nucleosome positioning, histone–DNA stoichiometry, and the degree of chromatin compaction define the transcriptional level of the DNA^5^ and have a wide-ranging influence on cell and organism functioning, including, e.g., ageing^6^.

Two dynamic interaction types within megabase chromatin are nucleosome clutches (clusters)^7,8^ and topologically associating domains (TADs)^9^. Three to ten neighboring nucleosomes on a DNA strand flanked by NFR form nucleosome clutches, and such clusters of nucleosomes may interact even if separated far apart^10,11^, with a large number of nucleosomes forming chromatin domains^12^. Genome-wide nucleosome positioning studies demonstrated that eukaryotic chromatin generally is heterogeneous without regular nucleosome positioning^1^, exhibiting such clutches formed by heterogeneous groups of nucleosomes^10,13^. A recent modeling study suggested that nucleosome-nucleosome interactions are essential for the formation of clutches, and the length of NFRs profoundly affects clutch size^14,15^. TADs arise from chromatin interactions within tens or hundreds of kilobases. While chromatin looping may occur within a TAD, active and inactive chromatin compartments may include several TADs^16,17^. TADs are suggested to represent local chromatin structures in interphase nuclei^16,18^.

Therefore, chromatin in vivo can be imagined as an array of irregularly positioned clusters of nucleosomes that may not condense into the 30-nm fibers observed by reconstituted nucleosome arrays obtained from the Widom ‘601’ DNA high affinity nucleosome positioning constructs commonly used to investigate chromatin in vitro^19^. In vivo studies instead suggest that chromatin condenses as interdigitated 10-nm fibers^20–22^. Furthermore, recent data shows that regular nucleosome spacing and DNA-sequence dependent positioning are not observed in vivo*,* even for the artificial ‘601’ positioning sequences assembled into the genome using the CRISPR/Cas9 technique^23^. Hence, there is a need to establish and characterize in vitro models of condensed TAD-sized chromatin fibers.

We have previously demonstrated^24–27^ that T4 DNA (~ 166 kbp) with natural nucleotide sequence can be reconstituted to chromatin fibers with controlled nucleosome occupancy and used as in vitro model of long TAD-sized chromatin fibers, suitable for single-molecule studies. λ-phage DNA (~ 48.5 kbp, λ-DNA) chromatin displayed similar properties as T4 DNA chromatin^26^. The size of the resulting λ-DNA chromatin allows the overall compaction dynamics to be addressed using both single molecule fluorescent microscopy (FM), electron microscopy (EM), atomic force microscopy (AFM), and single molecule force spectroscopy. These methods enable the nucleosome occupancy of individual fibers as well as nucleosome-nucleosome interactions and the formation of nucleosome clutches and domains to be quantified.

Since the pioneering observations of the double-helical DNA overstretching transition (formation of the so-called S-form)^28,29^, λ-DNA has become a convenient object in single molecule force spectroscopy studies. The DNA elastic properties^30,31^, the DNA interaction with molecules causing DNA extension (intercalating drug or recombinant RecA protein^32^), bending^33^, loop formation^31^, or condensation^34–36^ were investigated using the λ-DNA template. A number of single-molecule studies addressed the properties of the chromatin formed on the λ-DNA by applying the chaperone-assisted reconstitution method^37–40^. These papers investigated events of DNA unpeeling associated with the complete release of the DNA from the histone cores or related to the forced unwinding of the last DNA turn. However, the earlier work did not study the low-force (below 10 pN) behavior of nucleosome arrays on long DNA (like λ-phage). This force regime is particularly relevant for the folding/unfolding of higher-order chromatin structures, including the formation of regular fibers and topologically associating domains.

Though ‘601’ arrays and λ-DNA form suitable substrates for highly regular small nucleosome arrays and larger fibers, both are not natural substrates for nucleosome folding. It is an open question how this affects the stability of the formed nucleosomes, particularly their higher-order folding. Histone octamers have distinct sequence preferences^41,42^, as exploited in the ‘601’ template. Although λ-DNA features a wide sequence variety, it cannot be excluded that eukaryotic genomes have evolved sequence properties that direct nucleosomes to particular places, which has been speculated to mediate specific regulatory functions in vivo^43^. Therefore, it is imperative to validate features found on unnatural nucleosome DNA substrates by comparing these to native eukaryotic DNA. Although linker histones participate in the chromatin structure in higher eukaryotes, it has been shown that the viability of the cultured cells is resistant to significant H1 depletion^44^. In addition, complete linker histone knockout does not change the phenotype and transcription profile of the lower eukaryotes^44^. It has been found that the “native” folding of chromatin fibers can be reached without linker histone at millimolar Mg^2+^ concentrations^45,46^. These observations warrant the relevance of studying the nucleosome arrays without the linker histones.

Here, we have characterized in vitro nucleosome fibers reconstituted on different long native natural DNA and artificial substrates at a single molecule level using FM, negative stain EM, AFM, and force spectroscopy. Most importantly, we demonstrate the spontaneous tendency of TAD-sized chromatin to form nucleosome clusters.

## Results and discussion

### Nucleosome arrays on λ-DNA compact into dynamic clutches upon addition of Mg^2+^

**Figure 1:**
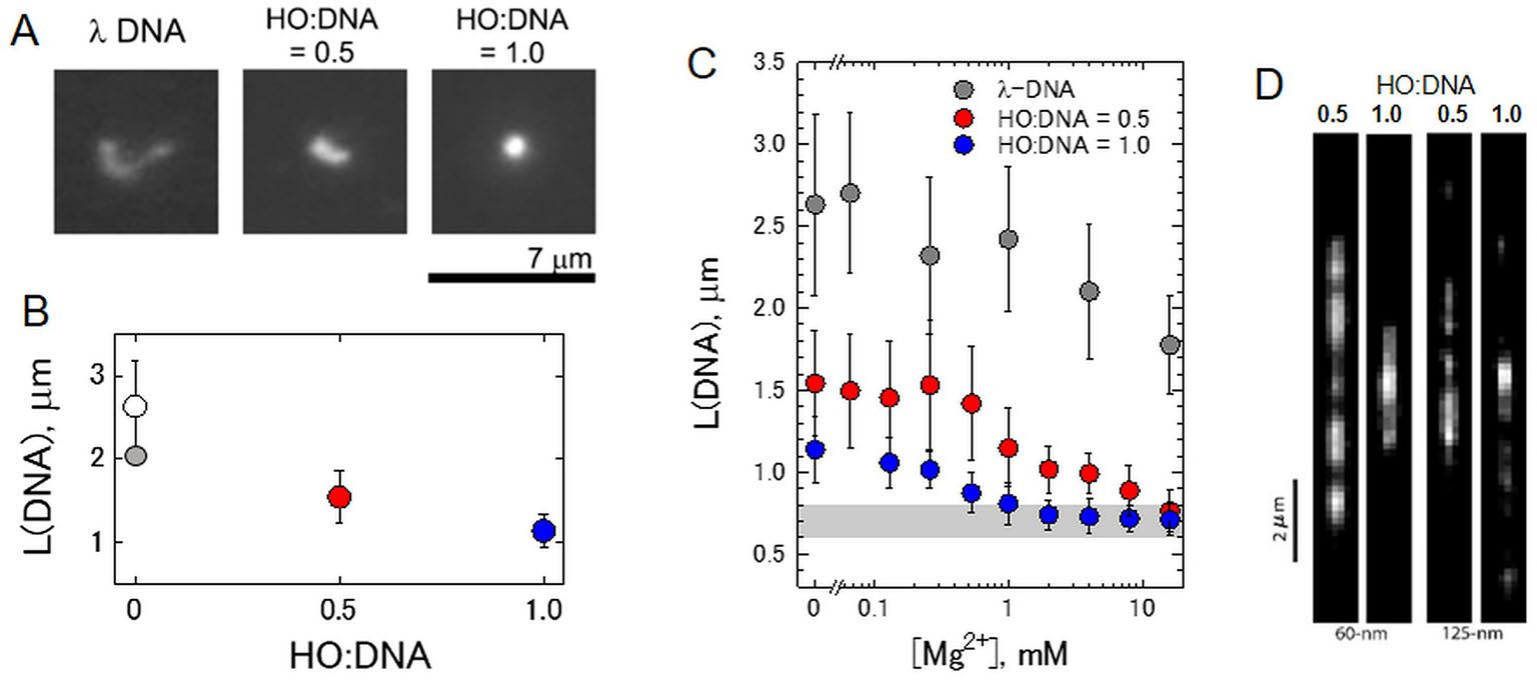
Fluorescence microscopy of reconstituted chromatin on λ-DNA show Mg^2+^-dependent compaction into dynamic clusters. (**A**). Typical fluorescence micrographs of a YOYO-labeled single λ-DNA molecule (left) and the nucleosome arrays reconstituted with 0.5 (center) and 1.0 (right) HO:DNA ratios. (**B**). Average long-axis length of λ-DNA and λ-DNA reconstituted with HOs as a function of a loading degree. The error bars indicate the standard deviations of the average values measured of *ca.* 100 individual DNA molecules or chromatin fibers. (**C**). Mg^2+^-induced compaction of λ-DNA and reconstituted nucleosome arrays. Changes in the average long-axis length of **λ-**DNA and nucleosome arrays at 0.5 and 1.0 HO:DNA ratios in the solution containing different concentrations of MgCl_2_. The error bars indicate the standard deviations of the average values measured of *ca.* 100 individual λ-DNA molecules or chromatin fibers. Shadowed areas indicate the long-axis length corresponding to the globular DNA conformation (0.6–0.8 μm). All solutions contain TE buffer with 100 mM NaCl. (**D**). Fluorescence still images of the λ-arrays in nanofluidic channels with HO:DNA ratios 0.5 and 1.0 in the 60-nm and 125-nm channels (see Supplementary Movies 1–4). OriginPro software^75^ (http://www.originlab.com) was used to create the graphs in (**B**) and (**C**).

To visualize the condensation of TAD-sized chromatin domains, nucleosome arrays on λ-DNA were labeled with YOYO-1 fluorescent dye. Bare λ-DNA and nucleosome arrays at HO:DNA ratios 0.5 and 1.0 exhibit free Brownian motion in solution (Fig. 1A). Increasing the HO:DNA ratio resulted in a progressive decrease of the long-axis linear dimension of the fibers from *ca.* 2.5 μm to 1.0 μm reflecting the formation of nucleosome arrays and their compaction at 100 mM NaCl (Fig. 1B).

The effect of Mg^2+^ on the conformational state of chromatinized λ-DNA was studied by varying the concentration of MgCl_2_ from 0 to 15 mM in TE buffer (10 mM Tris–HCl, 1 mM EDTA, pH 8) with added 100 mM NaCl (Fig. 1C). According to polyelectrolyte theory^47,48^, divalent cations cannot induce DNA conformational transition into a globular state due to insufficient DNA charge neutralization by weakly bound divalent counterions. Accordingly, increasing MgCl_2_ concentration up to several millimoles does not significantly affect the average long-axis length of λ-DNA coils (Fig. 1C). At higher Mg^2+^ concentration, a 20% decrease of the λ-DNA average long-axis length was observed, which can be explained by increasing the DNA flexibility caused by the electrostatic screening of DNA negative charges by the divalent cations. This globular state of DNA corresponds to tightly packed DNA condensates with an outer diameter of typically 100–200 nm^49^, nearly independent of DNA length, condensation conditions, and condensing agent. These DNA globules were observed by FM as ~ 0.6–0.8 μm moving particles due to the blurring effect (+ 0.3 μm in each dimension)^50^.

The condensation of the arrays at HO:DNA ratio 0.5 was more strongly affected by Mg^2+^, and the long-axis length started to shrink at < 1 mM Mg^2+^. The long-axis length at 15 mM of Mg^2+^ was only slightly larger than the typical size of DNA in the globular state (Fig. 1C). The saturated nucleosome arrays (HO:DNA = 1.0) progressively shrunk when only 0.1 mM of Mg^2+^ was added, and a complete conformational transition into a compact globular state was accomplished at *ca.* 2 mM of MgCl_2_ (Fig. 1C).

The essential difference between DNA and nucleosome arrays is the compaction at sub-millimolar and millimolar concentrations of divalent cations, in contrast to DNA that undergoes only modest contraction at [Mg^2+^] > 3 mM. Mg^2+^ promotes attractive nucleosome–nucleosome interactions^45^ predominately by an increased screening of the DNA charge and due to ion-ion correlation^51^ combined with histone tails bridging^52^. The observed difference is similar to the behavior observed for compaction of the longer T4 DNA chains (*ca.* 166 kbp) and corresponding reconstituted chromatin fibers reported in our previous studies^25,27^. It was earlier demonstrated that a significant HO loading (HO:DNA > 0.7) is necessary for the condensation of short nucleosome arrays^53^. However, we observed that even unsaturated arrays of long chromatin molecules undergo notable compaction by Mg^2+^ (Fig. 1C and^25^), probably due to loops formed between nucleosomes that mediate attractive interactions. Note, that compaction due to the stacking of neighboring nucleosomes of the saturated short 12-mer nucleosomal arrays^54^, and also in long λ-arrays (Fig. 1C) and T4 DNA-based arrays^25^, is completed in a very similar range of Mg^2+^ concentrations (2–4 mM). This similarity suggests that, despite the difference in the compaction geometry of short^54^ and long^25^ arrays, their phase transition is primarily determined by the same overall charge neutralization of macromolecular chains.

Next, we directly imaged the clustering of nucleosomes in sub-saturated arrays using FM imaging in the nanofluidic channels. Figure 1D shows still images from recorded videos (Supplementary Movies 1–4). Due to the sub-micrometer confinement, nanofluidic channels forced the array into a more elongated, quasi-1-dimensional state, making it possible to better observe the spatial density of the DNA compaction. An increase in the HO:DNA ratio resulted in progressive DNA compaction. The fibers appeared as bright blobs, stretched to a few micrometers, and connected by barely visible strands of uncompressed DNA. These blobs continuously changed shape and stochastically associated and dissociated. Accordingly, the fibers fluctuated between relatively open and more condensed configurations, which we attribute to the dynamic fluctuation in the condensation state of the relatively nucleosome-free regions of uncompressed DNA between clusters of nucleosomes that appear as blobs. No significant sticking of the nucleosome fibers to the channel substrate or coverslip was observed. The qualitative behavior of the fibers was similar in the 60-nm and 125-nm channels, though the molecules were stretched more in narrower channels. Previously, similar results were reported for chromatin reconstituted with bacteriophage T4-DNA^27^. The primary reorganization process is the association and dissociation of distant clutches of nucleosomes, mediated by the looping of the connecting DNA and chromatin. However, even in nanofluidic channels, FM does not have sufficient resolution to resolve these structures.

### Single-molecule force spectroscopy on large, heterogeneous chromatin fibers

Complementary to the FM, we used multiplexed magnetic tweezers (MMT) force spectroscopy to characterize the compaction and mechanical properties of the arrays reconstituted on the native human DNA, λ-DNA, and Widom ‘601’ nucleosome positioning sequence. This gives an independent assessment of individual fiber composition and compaction under similar physiological conditions as the FM at varying degrees of nucleosome occupancy. DNA templates for magnetic tweezers measurements (labeled by digoxigenin and biotin at the ends) were prepared and characterized as described in the “Materials and methods” section (Figs. S1 and S2). Nucleosome arrays were prepared by salt dialysis from the DNA and human histone octamers (see “Materials and methods”, Fig. S3).

The MMT measurements were carried out under physiological conditions with cations’ concentrations (100 mM K^+^, 10 mM Na^+^, 2 mM Mg^2+^) known to favor the formation of compact chromatin in the arrays reconstituted with the ‘601’ positioning sequence^20,52,55^. Our FM measurements clearly showed complete compaction of the λ-arrays under physiological conditions at HO:DNA = 1.0 with 2 mM Mg^2+^, while at HO:DNA = 0.5, the fibers appear not maximally but partially folded (Fig. 1A,C).

**Figure 2:**
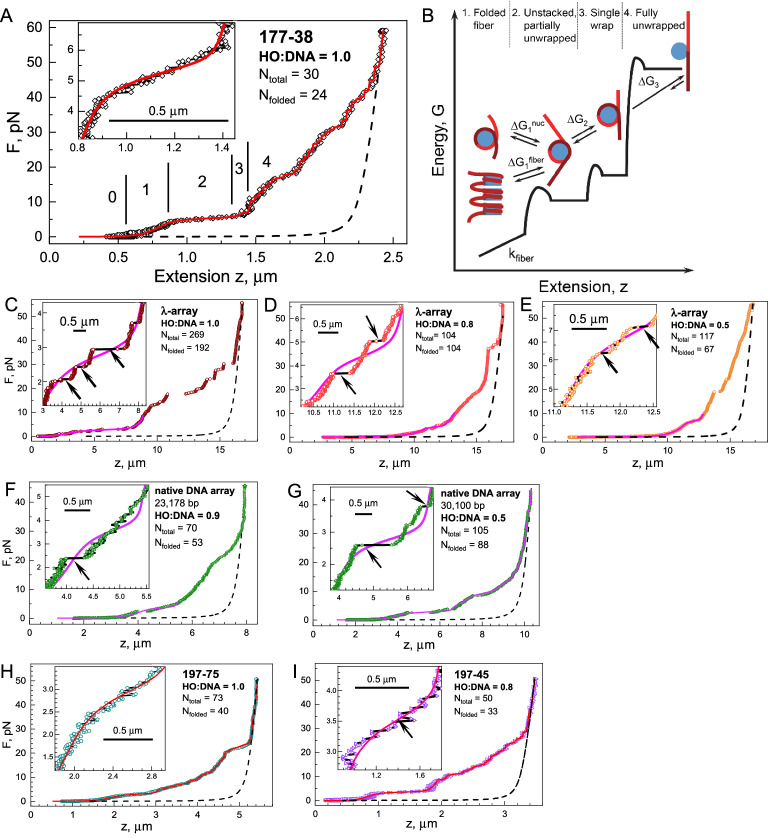
Single-molecule force spectroscopy reveals not only unstacking and unwrapping of nucleosomes in chromatin fibers but also large rupture events indicative of trans-interactions between remote parts of the chromatin fiber. (**A**). Example of an experimental stretching curve. Points are recorded data; the red line shows the model fitting. Different stages of the fiber stretching are indicated with numbers referring to the respective transition shown in (**B**). “0” indicates the extension of the bare DNA. (**B**). Statistical mechanics model for the single-molecule nucleosome array stretching. Free energy—extension scheme illustrating different stages of the nucleosome array extension under the influence of the stretching force. Deformation of the fiber includes (1) extension of the folded array; (2) transition of the array from a fiber to a bead-on-a-string chain accompanied by nucleosome unstacking and partial DNA unwinding; (3) deformation of the nucleosomes with further DNA unpeeling and possible dissociation of the histone dimer(s); (4) largely irreversible one-step rupture of the last turn of the DNA wrapped on the histone core. A detailed description of the model is given in the Material and Methods section. (**C**–**E**) λ-arrays form heterogeneous nucleosome clusters. Sample curves of the λ-array stretching recorded for the HO:DNA ratios 1.0 (**C**), 0.8 (**D**), and 0.5 (**E**). (**F**,**G**) native DNA array at HO:DNA ratio 0.9 (**F**) and ratio 0.5 (**G**). **(H**,**I)** Stretching curves of the arrays reconstituted on the ‘601’ positioning DNA template: (**H**) 197-75 array; (**I**) 197-45 array**.** Inserts show low-force regions corresponding to the stretching of the compacted arrays. Arrows indicate cluster–cluster ruptures. In each panel, the horizontal bar corresponds to 0.5 μm. Points connected by the lines are experimential data, smooth curves are statistical mechanics model fitting, and arrows indicate cluster–cluster ruptures. Dashed lines show the extension of the bare DNA calculated using the DNA contour length and the WLC model. OriginPro software^75^ (http://www.originlab.com) was used to create the graphs in (**A**) and (**C**–**I**). Microsoft PowerPoint 2016 (http://www.microsoft.com) was used to draw the image in (**B**).

As opposed to stretching of bare DNA, which produced reproducible curves in excellent agreement with the known mechanical properties and contour length of the DNA and fitted well with a worm-like chain (WLC) model (Fig. S2C), nucleosome arrays displayed a wide variation of unfolding events, similar to reports in other works (see, e.g.^56–58^). This observed inhomogeneity should be attributed to the variation in nucleosome number and was determined for each individual fiber. Still, despite these variations, the successive steps of unfolding stretching curves of the nucleosome arrays display regular features that can be captured in a statistical mechanics model^56,59,60^ developed for short, well-defined ‘601’ arrays (Fig. 2B). After elastically stretching the fiber assigned to the extension of the bare DNA (indicated “0” in Fig. 2B), a force plateau is observed at forces between 2 and 5 pN. The plateau extension is attributed to the unfolding of the fibers due to the rupture of nucleosome-nucleosome contacts and the partial release of the DNA from the histone octamer.

At around 7 pN, the nucleosomes gradually yield more DNA until each nucleosome wraps 77–80 bp of DNA. Next, the stepwise release of this last turn of the DNA superhelix is observed as discrete 25–27 nm steps, corresponding to 77–80 bp, at forces > 10 pN. This characteristic feature has been studied in many single-molecule measurements of nucleosome array stretching (see, e.g.^39,61,62^ and references cited therein) and allows reliable determination of the total number of nucleosomes in a given fiber (*N*_*total*_). The number of nucleosomes obtained this way is generally larger than the corresponding level of condensation below the plateau region. Careful analysis of short, regular ‘601’-based fibers concluded that not all nucleosomes contribute to the folding of the fibers at low force and we, therefore, assign the number of folded nucleosomes (*N*_*folded*_) separately based on the expected DNA nucleosome repeat length (NRL) and the array extension at F < 3 pN^56,59^.

We investigated the mechanical properties of the long chromatin fibers reconstituted on the λ-DNA and MNase-digested native DNA. Figure 2C–E show the force-extension curves of λ-DNA reconstituted at HO:DNA ratios of 1.0, 0.8, and 0.5. HO:DNA ratios were calculated based on a density of one histone octamer per 197 bp. All stretching curves of the λ-DNA array showed similar features as those of short regular nucleosome arrays (see Fig. 2A).

However, in addition to these common features shared with shorter chromatin fibers, we also observed large unfolding events at forces below 10 pN that were not observed previously. As exemplified by the arrows in the insets of Fig. 2C–E, such rupture events exceeded the Brownian fluctuation of the tether and yielded step sizes of up to several µm. In the shorter fibers, we did not observe such dramatic rupture events. We checked that large steps were not accompanied by lateral displacements of the bead, which are characteristic of breaking non-specific sticking of the fiber to the surface. These large rupturing events should therefore be attributed to the disruption of intra-fiber interactions, and the step size corresponds to the size of the chromatin loop released upon rupture of distant nucleosome-nucleosome interactions.

### Force spectroscopy on chromatin fibers reconstituted on native DNA

We complemented studying the λ-arrays with force spectroscopy measurements of chromatin fibers reconstituted on the MNase-digested native DNA (Fig. 2F,G). In these native chromatinized DNA fragments, we observed similar rupture events at a force below 10 pN. For comparison, Fig. 2H,I display representative force-extension curves recorded for arrays reconstituted on the ‘601’ positioning sequences with NRL = 197 bp. The native-DNA arrays share similar features in the force-extension curves as those on λ-DNA. Note that the length of each DNA template is unknown, as opposed to that of the λ- and ‘601’ arrays, resulting in arbitrary maximal extension of the fibers. To allow for a quantitative comparison of the curves, and detailed analysis with our statistical mechanics model for chromatin unfolding, we fitted the contour length of the free DNA, at F > 40 pN, where all nucleosomes are fully unwrapped, to the WLC model using the known DNA persistence length of 50 nm and stretch modulus of 1500 pN, shown as dashed lines. The resulting distribution of contour lengths roughly matches the size obtained by the electrophoretic mobility shift assay (EMSA), with a mean of about 10 kb and a wide distribution between 0.5 and 40 kb. Further characterization of the native template and validation of the fits is given in Supplementary Fig. S2C,D.

Using the obtained contour length for the arrays reconstituted on native DNA made it possible to compare their mechanical properties with those of the λ-arrays and ‘601’ arrays. We observed the same phenomena qualitatively: the unfolding was well-captured by the model, except for the exemplary large rupture events at forces below 10 pN. These rupture events occurred far less frequently on the ‘601’ arrays (e.g. Fig. 2I), but it should be noted that the shorter length of the ‘601’ templates would, given the same statistical occurrence, yield both fewer rupture events and also smaller rupture steps. The low-force parts of the curves for most of the 197-45, 197-75, and 177-38 arrays show that the unfolding was a gradual process consisting of short reversible steps (e.g., inserts in Fig. 2A,H,I). Additional data illustrate that this cluster–cluster ruptures are occasionally observed for arrays with NRL = 197 bp and predominantly seen at HO:DNA = 0.5 (Figs. S6 and S7A). For most traces, though, the fluctuating short steps in the plateau region of the nucleosome arrays reconstituted on the ‘601’ DNA templates appear homogeneous, and their fitted curves overlay with the experimental data. We also studied arrays with NRLs 177, 172, 166, and 162. These arrays were reconstituted at HO:DNA ratios close to stoichiometry 1.0. Sample stretch-relief data of each type of array and their fitted curves are shown in Fig. S4. Figure S5 shows the distribution of numbers of the total and folded nucleosomes of the studied arrays.

Though we could produce and measure longer ‘601’ arrays by ligation, a complication of this approach is that it resulted in a mixture of three arrays: for the 197 array, we obtained mono-, tri-, and pentamers, with 197-15 arrays present in larger amounts than the 197-45 array and the 197-75 array being a minor fraction. Only 7 traces were recorded for the 197-75 arrays (one is shown in Fig. 2H), and we do not give statistics for this array.

We obtained many more curves of the λ- and long native-DNA arrays, and the plateau regions generally featured a combination of gradual and abrupt unfolding steps (inserts in Figs. 2A,C–G, S6, and S7). This suggests that the large compacted but sub-saturated fibers consist of nucleosome clusters compacted into intra-molecular loops and separated by stretches of bare DNA or sparsely distributed nucleosomes.

### EM, AFM, and single molecule stretching reveal loops of irregular clusters of nucleosomes on long chromatin fibers

**Figure 3:**
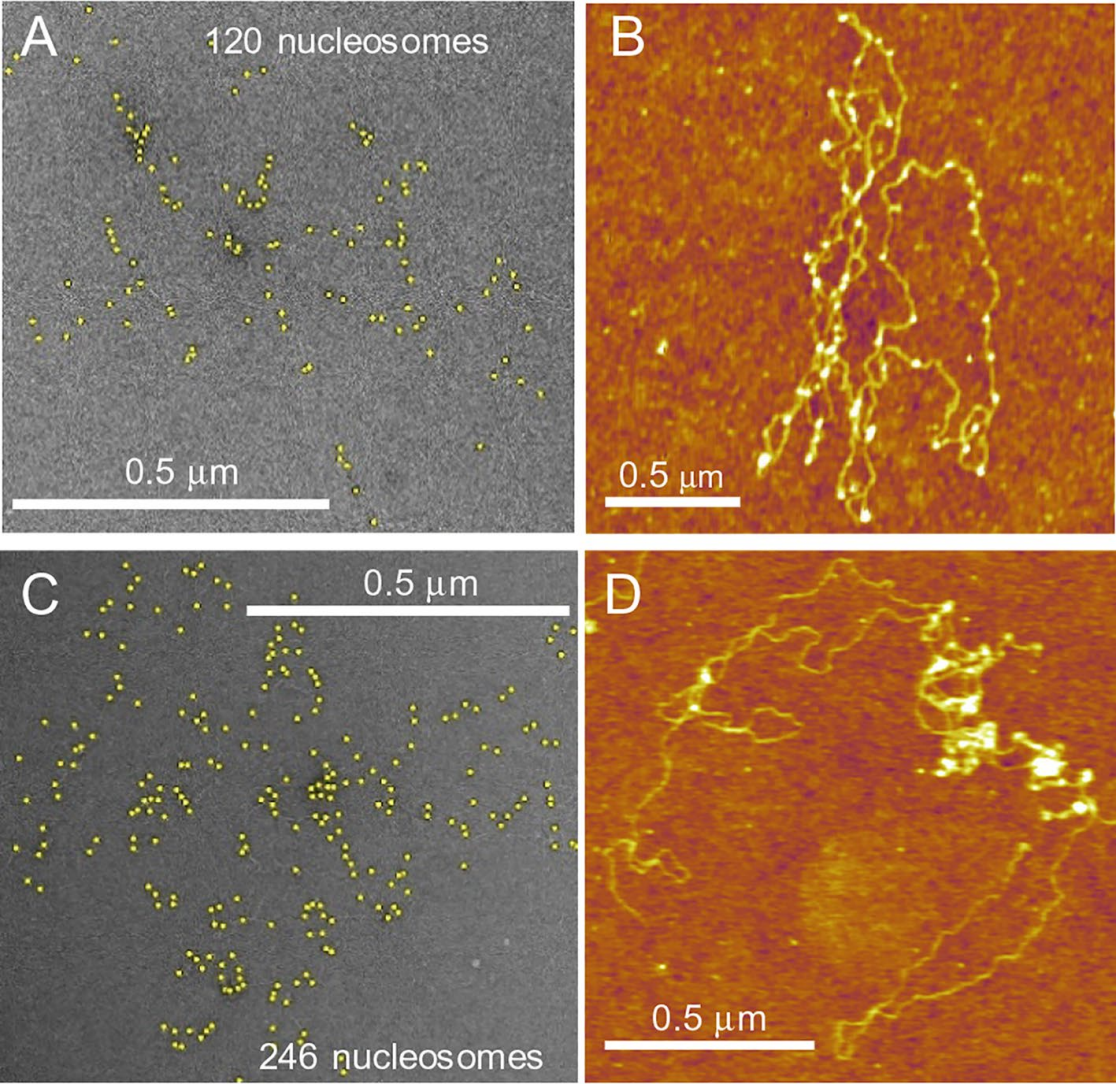
EM and AFM images illustrate the formation of heterogeneous nucleosome clusters in the λ-arrays. (**A**–**D**) Images of the λ-DNA arrays obtained for the HO:DNA 0.5 (**A**,**B**) and 1.0 (**C**,**D**) by the negative staining EM (**A**,**C**) and the AFM (**B**,**D**) methods.

**Figure 4:**
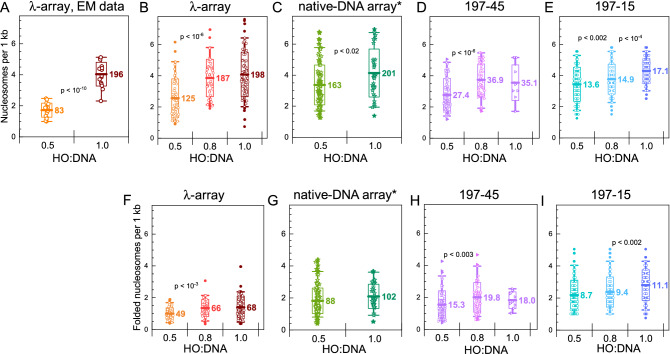
Nucleosome count in the arrays reconstituted on the λ-DNA (**A**,**B**), MNnase-digested genomic DNA (**C**), and 197 bp NRL ‘601’ DNA templates (**D**,**E**). The relative number of nucleosomes (per 1 kb of the DNA template) in dependence on the HO:DNA ratio is shown for the EM images (**A**), for the MMT measurements of the λ-array (**B**), native DNA array (**C**), 197-45 (**D**), and 197-15 arrays (**E**). For the EM data in (**A**), nucleosomes were counted in the 17 arrays for each HO: DNA ratio. (**F**–**I**) Show the dependence of the number of nucleosomes in the folded domains of the arrays on the DNA ratio determined by the MMT method. Mean values of the nucleosomes are displayed in the graphs. *For the native-DNA arrays in which the DNA lengths varied in a broad range, the observed values *N*_*total*_ (**C**) and *N*_*folded*_ (**G**) were projected to an imaginary DNA length equal to that of the λ-DNA: *N*_*projected*_ = *N*_*observed*_·(48,548 bp/*L*_*DNA,observed*_). If a pair of mean numbers have a significant statistical difference, the respective p-value is displayed. In the graphs, thick lines with labels indicate mean value; boxes show range ± standard deviation; whiskers show 5–95% range of data; solid points indicate outliers. Numerical data are given in Supplementary Table S3. OriginPro software^75^ (http://www.originlab.com) was used to create the graphs.

Though MMT can probe nucleosome-nucleosome interactions at low force and with high precision, it does not provide any information on the topology of the folded fiber. Therefore, we imaged the λ-arrays at the HO:DNA ratios 0.5 (Fig. 3A,B and Fig. S7F) and 1.0 (Fig. 3C,D and Fig. S7G–I) with negative stain EM (Fig. 3A,C and Fig. S7F, G) and AFM (Fig. 3B,D and Fig. S7H, I). Both methods are capable of imaging with nanometer resolution, but the different contrast mechanisms and sample preparation give complementary information. All images feature loosely folded long DNA molecules with small globular features corresponding to single or multiple nucleosomes. The DNA is entangled and held together by clutches of nucleosomes at the crossings for both HO:DNA ratios. In addition, the nucleosomes are not evenly spread over the DNA but appear somewhat clustered. The EM micrographs resolve the individual nucleosomes better, and we collected statistics on the number of nucleosomes per λ-DNA as a function of the HO:DNA ratio (Fig. 4A). Note that not all particles have the same size, and the angle at which the DNA exits the nucleosomes suggests that in many cases, we observed sub-nucleosomal particles that could be present after reconstitution or could have formed during sample preparation before imaging. In addition, it is unavoidable that we could miss counting some nucleosomes within and beneath the aggregate clusters. So similar to our observations with force spectroscopy, EM and AFM imaging displayed a mixture of folded and partially folded nucleosomes. Nevertheless, the topologically intertwined, looped structures imply a DNA condensation mechanism mediated by interactions between distant nucleosomes.

### Quantification of nucleosome loading on diverse DNA templates

Whereas the use of ‘601’ positioning elements allows for reconstitution of designer chromatin fibers with excellent control over the number and positions of the nucleosomes, this artificial DNA sequence prohibits the study of biologically relevant DNA templates. It may obscure relevant structural aspects dependent on DNA sequence or irregular spacing of nucleosomes, like the formation of clutches and loops. We used EM and MMT methods to quantify the reconstitution yield of various DNA substrates under identical conditions. Figure 4A,B show the results of nucleosome counting of the λ-array with an increasing HO:DNA ratio. At HO:DNA = 0.5, EM yields a lower nucleosome count than MT, but at HO:DNA = 1.0, we obtained a very similar number. This discrepancy between EM and MMT data at a low HO:DNA ratio can be attributed to some nucleosome loss during the EM sample preparation and the smaller statistics for the EM dataset. Apparently, nucleosomes are better stabilized in denser chromatin fibers. This suggestion was confirmed when limiting the analysis to only fully folded nucleosomes (Fig. 4F): the more nucleosomes on the DNA, the more nucleosomes were observed to be fully folded.

Following the increased HO:DNA ratio, we found 125 and 198 nucleosomes for half and fully saturated fibers. Thus, the DNA was highly saturated at a 1.0 ratio of HO:DNA. Interestingly, however, the numbers of fully folded nucleosomes were only 49 and 68, meaning that 39% and 34% of the nucleosomes were folded.

It is interesting to compare the reconstitution yield on λ-DNA with that on ‘601’-arrays (Fig. 4D,E,H,I). The MMT method allowed unprecedented statistics—in total, about 3300 array traces were selected and analyzed using the statistical mechanics model^59^ (Tables S3 and S4). As well-established before, we obtained a high loading yield on the ‘601’ arrays, though the larger arrays containing 197-45 repeats did not assemble as well (78%) as the shorter 15 repeats (114%) at HO:DNA ratios of 1.0. The fraction of folded nucleosomes was also lower in the 197-45 arrays (57%, 54%, 51%) than in the 197-15 arrays (64%, 63%, 65%) for HO:DNA ratios of 0.5, 0.8 and 1.0, again pointing to increased nucleosome folding in denser fibers.

As observed before^56^, we detected quite a bit of heterogeneity within a reconstituted batch, which could either be caused by variations in reconstitution yield or disassembly during sample preparation of the flow cell. A reconstitution yield above 100% can be achieved in the 197-45 and 197-15 arrays because these templates contain 1005 bp additional DNA in the handles flanking the ‘601’ sequences. Additional nucleosomes are expected to form on the handles when the HO:DNA ratio exceeds 1.0. On the other hand, the absence of positioning signal in λ-DNA is expected to lead to a primarily stochastic nucleosome distribution during the initial stages of the in vitro array reconstitution and may prevent the formation of saturated arrays with a stoichiometric number of nucleosomes.

However, nucleosome assemblies on both λ-DNA and ‘601’-based arrays cannot be found in nature, and the specific sequences of these templates may obscure relevant differences with eukaryotic DNA. Indeed, we observed different loading yields when we reconstituted chromatin fibers on random sequence native DNA obtained from MNase-digested human DNA (Fig. 4C,G). At sub-stoichiometric HO:DNA ratios, the number of nucleosomes per 1 kbp was substantially larger than observed for the λ-arrays and closer to the 197-45 and 197-15 arrays (3.4 versus 2.6, 3.1, and 4.6, respectively). At an HO:DNA ratio of 1.0, the reconstitution yield was similar for all long templates (λ-DNA, native-DNA, and 197-45), though not as high as for the short 197-15 array (4.1 versus 4.1, 4.0 and 5.8). This trend was also present when only confining the analysis to folded nucleosomes (Fig. 4F–I). Thus, the native DNA has a higher affinity for histones than λ-DNA.

### Analysis of ruptures in condensed fibers

In the compacted inhomogeneous arrays, one would expect that the attractive forces between the inter-cluster contacts and nucleosome-nucleosome interactions inside the clusters have similar molecular features and strengths. Both folding of the 10-nm beads-on-a-string fiber and cluster–cluster interaction are facilitated by reducing the DNA-DNA repulsion due to charge screening by the positively charged histone tails, Mg^2+^, and Na^+^/K^+^ ions. Histone tail bridging and attractive counterion-DNA charge-charge correlations also contribute to DNA-DNA attraction^52,63^. The unfolding of inhomogeneous but compact chromatin structures proceeds as a combination of loss of cluster–cluster contacts and nucleosome-nucleosome contacts within the clusters. To evaluate the low-force ruptures unbiasedly, we wrote a Python script that automatically identifies steps larger than 200 bp (roughly the amount of DNA released upon unwrapping a single nucleosome). Ruptures shorter than 200 bp are not distinguishable from the step-wise unfolding of the fiber occurring at low forces (1 to 6 pN; Figs. 2 and S6) and/or thermal fluctuations. These events could consist of the rupture interactions of nucleosomes that are close but further apart than the stacking nucleosomes in regular, 601-based two-start or one-start 30-nm fibers. Because of the small rupture length, these transitions would not be captured in the statistical mechanics model. However, the similarity of the fitted model parameters (in particular (*k*_*fiber*_) and *∆G*_*1*_) in all types of fibers suggests that stacking interactions of such more remote nucleosomes have a relatively small effect.

**Figure 5:**
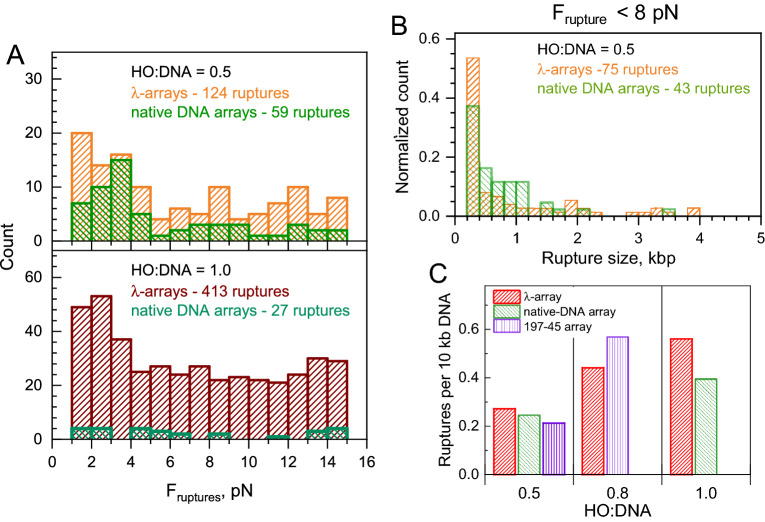
Analysis of the cluster–cluster ruptures in the λ-arrays, native DNA arrays, and 197-45 arrays. (**A**). Distribution of rupture force (*F*_*rupture*_) observed for the λ-arrays with HO:DNA ratios 0.5 (top, orange bars) and 1.0 (bottom, dark red bars) in comparison with the data obtained for the native arrays with similar HO:DNA ratios (green bars). The total number of ruptures recorded for each array type is indicated in the graphs. (**B**). Normalized distribution of the cluster–cluster distance determined for the λ-arrays for *F*_*rupture*_ < 8 pN. HO:DNA ratios and numbers of the observed ruptures are indicated on the graphs. The distance is expressed in kbp DNA. The data obtained for the λ-arrays at HO:DNA = 0.8 and for the 45-197 arrays HO:DNA = 0.5, 0.8, and 1.0 are presented in Fig. S9 of the Supplementary Data. (**C**). The number of the cluster–cluster ruptures normalized to 10 kb DNA using *F*_*rupture*_ < 8 pN cutoff and the number of the recorded traces at different HO:DNA ratios. The number of ruptures obtained for the 197-45 array at HO:DNA = 1.0 is not shown due to the small number of data (9 traces and 11 cluster–cluster ruptures). OriginPro software^75^ (http://www.originlab.com) was used to create the graphs.

We counted cluster–cluster ruptures and determined the length of the DNA release in each of these events (see “Materials and methods”) and the corresponding rupture force. A summary of the cluster–cluster ruptures’ analysis is presented in Figs. 5 and S8. Stretching curves of the individual λ-array fibers displayed at least one, but often two or three cluster–cluster rupture events (see examples in Fig. 2C–E). Similarly, stretching the relatively long native-DNA arrays displayed frequent cases of cluster ruptures (Figs. 2F,G, 5A,B). In contrast, only about 20% and 55% of the 197-45 arrays contain one cluster–cluster contact at HO:DNA 0.5 and 0.8. The shorter distance between most clusters (200–400 bp) makes them less visible in the experimental data (Fig. 2I). However, when normalized by the DNA length (λ-DNA is about 5 times longer than 197-45 DNA), the frequency of cluster–cluster ruptures is similar for all types of arrays (Fig. 5C). The relative frequency of the ruptures increases with the HO:DNA ratio (Fig. 5C). The native-DNA, λ-, and 197-45 array rupture statistics were collected from datasets of comparable size (Table S3). The native-DNA, shorter arrays, and 197-45 show significantly fewer rupture events (Figs. 5A and S8A), with most of the stretching curves of the 197-45 arrays not showing any ruptures at all. Cluster-rupture statistics collected for the native arrays at HO:DNA = 1.0 is less representative than the one for the λ-arrays (17 versus 242 hits). Still, we included the native-DNA array results and considered the rupture numbers 0.4 (native-DNA) and 0.56 (λ-array) per 10 kbp to be reasonably similar. Statistics collected for the 197-45 arrays at HO:DNA = 1.0 is poor (Figs. 5A and S8A) and is not analyzed here.

Forces at which clusters contacts rupture are distributed evenly in the range of 1–15 pN with somewhat higher frequency at the low and high forces (Figs. 5A and S8A). Assuming that all nucleosome-nucleosome interactions between clutches have the same strength, the broad distribution of rupture forces suggests that the number of nucleosome-nucleosome interactions varies widely, consistent with different sizes of nucleosome clutches. More ruptures at high force might be caused by a combination of cluster–cluster ruptures and unwrapping of the last DNA turn from the histone core. For stricter analysis, the data was split into two parts: ruptures at 1 < *F*_*rupture*_ < 8 pN (Figs. 5B and S8B) and 8 < *F*_*rupture*_ < 15 pN (Fig. S8C). The choice of 8 pN as a separation point was made to filter out last-turn nucleosome unwrapping events. However, a very similar distribution was obtained. Most ruptures have a 200–400 bp size, which can be explained by more frequent contacts between closer, neighboring regions of the arrays.

Higher saturation of the fibers resulted not only in longer plateau lengths but also in more rupture events. For the illustrative F-z curves shown in Fig. 2C–E, the plateau lengths are about 2.2 µm for HO:DNA = 0.5 (from 11.2 to 12.4 µm); 2.5 µm (HO:DNA = 0.8, from 10.2 to 12.7 µm); and 5 µm (HO:DNA = 1.0, from 3 to 8 µm). Additionally, the heterogeneity of the stretching curves in the plateau region increased at the higher HO:DNA ratios (see inserts in Fig. 2A–C). At HO:DNA = 0.5, the array unfolding looks similar to that observed for the shorter arrays (compare inserts in Fig. 2E,I, see also Figs. S6 and S7). At HO:DNA = 0.5, clusters of nucleosomes are separated by long stretches of DNA that do not interact with each other. For HO:DNA = 0.8 and 1.0, cluster–cluster aggregation leads to the formation of compacted fibers. This interpretation is supported by the EM and AFM images of the more open fibers at ratios of 0.5 (Fig. 3A,B) and more convolved structures at 1.0 (Fig. 3C,D).

The plateau region shifts to lower force with the increase of the HO:DNA ratio. In the plotted F-z curves, the plateau level shifts from 4.5–7 pN (HO:DNA = 0.5; Fig. 2E) to 2.5–6 pN (HO:DNA = 0.8; Fig. 2D) and 2–4 pN (HO:DNA = 1.0; Fig. 2C). The native-DNA arrays show similar behavior (Fig. 2F,G). This shift might be explained by the increase in the number of nucleosomes that stabilize cluster interactions in the folded aggregates.

In conclusion, the large steps in the force-extension curves at F < 8 pN should be attributed to the rupture of distant nucleosome cluster–cluster contacts, and these ruptures are irregular and vary over a wide range of lengths and contact strengths.

### The mechanical properties of the native-DNA, λ-, and ‘601’ 197 NRL arrays are similar

**Figure 6:**
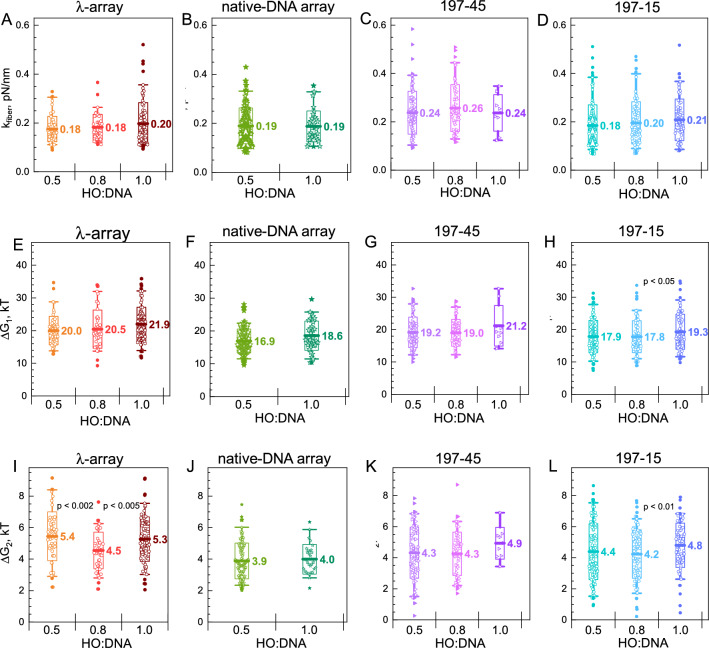
Mechanical properties of the λ-arrays, arrays reconstituted on the MNase-digested genomic DNA, positioned 197 bp NRL arrays obtained for different HO:DNA ratios by fitting experimental data to the statistical mechanics model. (**A**–**D**) stiffness (*k*_*fiber*_) of the folded arrays; (**E**–**H**) free energy of the nucleosome folding and unwinding of the first 53 bp of the DNA from the histone core (*∆G*_*1*_); (**I**–**L**) free energy of the further unwinding of the 13 bp DNA (*∆G*_*2*_). The left columns (**A**,**E**,**I**) is the data for the λ-arrays; second, the left column (**B**,**F**,**J**) is for the native DNA arrays; second, the right column (**C**,**G**,**K**) is for the 197-45 arrays; and the right column (**D**,**H**,**L**) is for the 197-15 arrays. In the graphs, thick lines with labels indicate the mean; boxes show the range within mean ± sd; whiskers show a 5–95% range of data; solid points indicate outliers. OriginPro software^75^ (http://www.originlab.com) was used to create the graphs.

The statistical mechanics model to determine the mechanical and thermodynamic properties of chromatin can still be applied to all arrays studied in this work, including the under-saturated fibers. Bare DNA segments folded into loops will not contribute to the extension and do not need to be taken into account. Bare fractions that do not form a loop, on the other hand, will add to the contour length of the free DNA and will therefore not affect the unfolding parameters, such as the stretching modulus of the array unfolding, *k*_*fiber*_, the free energy of transition from compacted fiber to bead-on-a-string structure, *∆G*_*1*_ and the energy of partial DNA detachment from the HO, *∆G*_*2*_. Figure 6 summarizes the distribution of these parameters for fibers with the variation of the HO:DNA content (λ-arrays, native-DNA arrays, 197-45, and 197-15 arrays). The results are tabulated in Table S3. The mean values of stiffness (*k*_*fiber*_) and unfolding energy (*∆G*_*1*_) increase slightly with the HO:DNA ratio increase, though this change is not statistically significant. We found a considerable spread between individual molecules from the same batch, but the variance is similar for the longer fibers compared to the short 197-15 arrays and agrees with previous findings on the ‘601’ arrays^58^. Thus the difference between the levels of saturation is predominantly in the number of nucleosomes that have been reconstituted (see Fig. 4), but clutches of nucleosomes within the fibers have similar mechanical properties.

**Figure 7:**
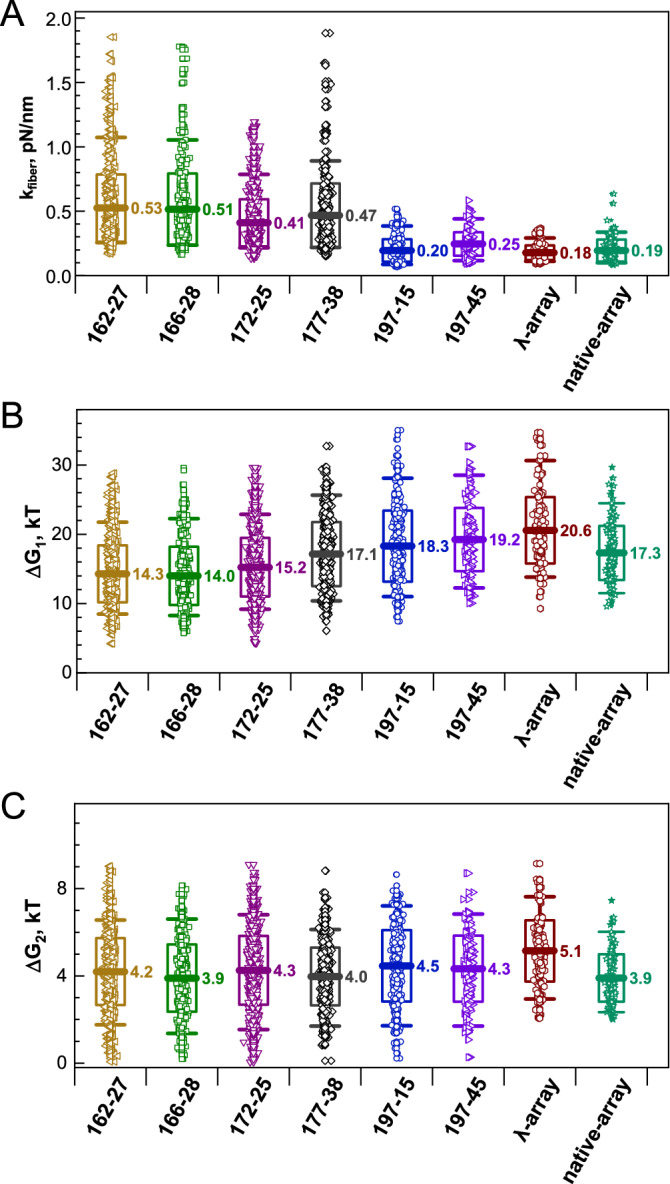
Summary of the mechanical parameters determined for the arrays studied in this work. Nucleosome array (**A**) stiffness (*k*_*fiber*_; the first stage of pulling), free energies of (**B**) the second (*∆G*_*1*_) and (**C**) third (*∆G*_*2*_) stages of the array pulling. For the arrays reconstituted on the ‘601’ positioning DNA, the nucleosome repeat length and number of the repeats are indicated on the x-axis. Mean values of the respective parameters are displayed in the graphs. Boxes show the range within the mean ± sd; whiskers show the range from 5 to 95% around the median value. OriginPro software^75^ (http://www.originlab.com) was used to create the graphs.

Since we did not observe differences as a function of HO:DNA ratio, we pooled the data for each type of array and compared averaged mechanical properties of the λ-, native-DNA to a range of ‘601’ arrays with different nucleosome repeat lengths (Fig. 7, Tables S3 and S4) for which we obtained a similar number of traces. The total number of ‘601’ fibers for comparison was about 3700. As reported before, in the NRL range from 162 to 177 bp, the stiffness of the arrays shows a slight decrease with an increase of the NRL: from *k*_*fiber*_ = 0.53 pN/nm (NRL = 162 bp to *k*_*fiber*_ ~ 0.41–0.47 pN/nm (NRL = 172 and 177 bp) and is significantly larger than that for NRL = 197 bp. This should be ascribed to the shorter linker length, which makes the compliance of chromatin fiber with unstacked nucleosomes less, or stacking the nucleosomes into a 2-start zig-zag fiber, which doubles the stiffness relative to a single stack of nucleosomes^58^. The parameters *k*_*fiber*_, *∆G*_*1,*_ and *∆G*_*2*_ determined in this work agree with previously published tweezers results of ‘601’-DNA arrays^56,58,64,65^, albeit showing slightly lower values. These minor discrepancies might be caused by the difference in the sequences in the linker DNA^58^ or by unaccounted differences in the experimental protocols applied in the different laboratories.

The native- and λ-DNA feature a stiffness comparable to that of the NRL = 197 bp arrays with a stiffness *k*_*fiber*_ ~ 0.18–0.25 pN/nm. The change of the energy associated with the unfolding of the array to the bead-on-a-string structure, *∆G*_*1*_, shows a gradual rise with the increase of the NRL (Fig. 7B), indicating that longer linker DNA can better accommodate DNA deformations that accompany nucleosome stacking. The native-DNA arrays display lower *∆G*_*1*_ values than the λ-arrays.

All arrays (based on the λ-, native-DNA, and ‘601’-arrays) show similar mean values of *∆G*_*2*_ in the range 3.9–5.2 kT with slightly higher (but within the data spread) numbers for the λ-arrays (Fig. 7C). This is expected as partial DNA unwrapping does not involve other nucleosomes and should thus be independent of the density of nucleosomes on the DNA. However, DNA sequence effects may differ between ‘601’ and non-‘601’ based nucleosomes.

The distributions of fiber stiffness (*k*_*fiber*_) all feature a small broadening at higher values as compared to a normal distribution (Fig. S9A,C,E,G), probably due to the significant heterogeneity in nucleosome positions in such fibers and the fact that the observed rupture events, that are not included in the model, reduce the fitted slope proportional to the rupture size. Nevertheless, the comparable *∆G*_*1*_ energies (Fig. S9B,D,F,H) show that the properties of the nucleosomes are independent of the fiber composition. In addition, *∆G*_*1*_ energies are in good agreement with the literature data for arrays with 197 bp NRL^56–58^. The excellent agreement of the mechanical parameters obtained from long and under-saturated fiber and the large rupture events at the low force that we report here should be attributed to the rupture long-distance nucleosome-nucleosome interactions rather than the cooperative disassembly of clusters of histone (sub)assemblies.

## Conclusions

Recent cryo-EM studies reported that in vivo nucleosome arrays lack even a short-range uniformity in the linker DNA lengths^13^, with nucleosomes displaying a wide range of breathing-DNA conformations^66^. Here, we report that in vitro reconstitution of nucleosome arrays on native-DNA and λ-DNA resulted in heterogeneous nucleosome spacing, which nevertheless form compact single-molecule aggregates consisting of clusters of folded arrays. Our data confirm that nucleosome arrays in natural DNA exist as dynamic irregular structures and that fibers reconstituted on these substrates may serve as a better model for chromatin in vivo than arrays reconstituted on the ‘601’ high affinity nucleosome-positioning DNA sequence. The dynamic reorganization observed in FM of fibers in nanochannels and the low and broadly distributed rupture forces that we measured with MMT also agree with experimental observations^20,22^ and a recent modeling study^67^ reporting a liquid-like dynamic chromatin state. That modeling work revealed that physiological salt conditions stimulate nucleosome “breathing” (dynamic winding-unwinding of the DNA from the histone core) while static DNA attachment to the HO prevails at low salt. This difference in DNA dynamics results in the perturbation of the regular fiber structure without losing its compactness. Loss of energy in rupturing static contacts is compensated by increased entropy and the formation of numerous new weak and dynamic bonds. The observed absence of abrupt changes of the mechanical properties (stiffness *k*_*fiber*_, energies *∆G*_*1,*_ and *∆G*_*2*_) in dependence of NRL, which is reported in this work and a recent detailed study^58^, supports the model of dynamic breathing chromatin structure.

An important finding of this work is that it is predominantly the reconstitution yield rather than the local structure of nucleosome clutches or mechanical properties of the fibers that defines the existence and frequency of the cluster–cluster contacts. We did not find that the difference in DNA sequence (artificial ‘601’ nucleosome-positioning, prokaryotic λ-DNA, or human DNA) makes a noticeable difference for cluster–cluster statistics.

The unfavorable contribution of bare DNA-DNA repulsion weakens the forces that hold the clusters together. At HO:DNA = 0.5, large stretches of bare DNA may not be included in the clusters of nucleosomes and contribute only to the bare DNA extension. A similar conclusion was earlier drawn based on stretching T4 chromatin fibers using a flow-stretching technique^24^, in which partially folded structures containing both unfolded and compacted globular regions of various sizes were observed. This was attributed to the heterogeneous distribution of nucleosomes on the DNA chain. The observed variance in the rupture forces and cluster–cluster distances is not the result of the low-resolution of the MMT measurements (the force accuracy is sub-pN, and the extension resolution is several nm) but is intrinsic to the mechanics of the reconstituted fibers.

The current methods for reconstitution, force spectroscopy, and data analysis may be used in further studies to test whether different chromatin fractions, such as heterochromatin or euchromatin, yield different reconstitution yields and/or folding properties. Our current results do not indicate that DNA sequence is essential in the higher-order folding of heterogeneous chromatin fibers.

The question arises why the in vitro salt-dialysis reconstitution of the native DNA and the λ-arrays results in inhomogeneous clusters of the nucleosomes rather than in an entropy-advantageous random distribution of the nucleosomes along the entire DNA length? A plausible explanation is that the entropy gain associated with a random distribution of the nucleosomes along the entire DNA length is counteracted by the favorable attractive nucleosome–nucleosome interactions. Under the conditions of the nucleosome array reconstitution, the decrease of salt concentration leads first to the formation of the tetrasomes (complex of the DNA and (H3/H4)_2_ tetramer) followed by consecutive addition of the two H2A/H2B dimers. At the moderate monovalent salt concentration (150–400 mM), nucleosome-nucleosome interaction is attractive^45,52^, favoring nucleosome array folding. Hence, closely separated nucleosomes can form clusters since the favorable contribution of the attractive forces overcomes the entropy factor facilitating random and relatively uniform nucleosome distribution. This finding is consistent with a cooperative mode of nucleosome reconstitution^68,69^. Folding of the nucleosome arrays is a specific case of the general phenomenon of DNA condensation. DNA condensation is a cooperative process^52,70,71^ which means that under the conditions when polycationic condensing agents (like histones in the living cells) are present in an amount that is insufficient for compaction of all DNA, there will be a coexistence of regions of condensed and bare DNA.

## Materials and methods

### Preparation of labeled DNA templates for tweezers measurements

(A detailed account of DNA preparations is given in the Supplementary Methods).

MNase-digested human DNA for multiplexed magnetic tweezers (MMT) measurements was prepared by limited digestion of nuclei from Expi293 cells (Gibco, ThermoFisher Scientific Inc.) followed by purification (Supplementary Methods) to extract native DNA of chromatinized origin. Next, two complementary digoxigenin-labeled DNA oligomers and two biotin-labeled DNA oligomers were dephosphorylated and then annealed to form double-stranded digoxigenin- and biotin-labeled fragments. Then the double-stranded oligomers were ligated to the human-source DNA with blunt ends using T4 DNA ligase (New England Biolabs) (Fig. S1A). Arrays were reconstituted with MNase-digested genomic DNA and human HO with HO:DNA molar ratio at 0.5, 0.9 and 1.0. λ-phage DNA for magnetic tweezer force spectroscopy measurements was prepared by ligating the two complementary digoxigenin-labeled DNA oligomers and two biotin-labeled DNA oligomers to the 12-bp sticky ends of λ-DNA using T4 DNA ligase (New England Biolabs) (Fig. S1B).

DNA templates with NRLs 197, 177, 172, 166, and 162 bp were prepared as detailed in the Supplementary Information (Supplementary Methods and Figs. S1–S3). In addition to monomers of the ‘601’ 197-15 array, DNA ligation also yielded multimers of ‘601’ arrays, which we exploited to measure larger regular nucleosome arrays (197-45 and 197-75). Only ligation products containing uneven multiples of ‘601’ arrays can be terminated with complementary digoxigenin and biotin and were observed in the tweezers (Figs. S1C, S2C, S3B).

The quality of the DNA labeling was tested by the MMT measurements and fitting of the experimental data to the worm-like chain (WLC) model (sample curves are shown in Fig. S2C). For known DNA lengths (e.g., 48,548 bp for the λ-DNA), the fitted values of the DNA persistence length (45–50 nm) and the DNA stretching modulus (900–1200 pN) are in perfect agreement with the well-established values for the double stranded DNA. The contour length of MNase-digested native DNA was fitted for each molecule. Most DNA fragments were in the range of 4–24 kbp, in agreement with the gel electrophoresis data (Fig. S2A).

### Electrophoretic mobility shift assay (EMSA)

The electrophoretic mobility shift of λ-DNA nucleosome array reconstituted with histone octamers was assessed by agarose gel (Fig. S3A). The equivalent of 200 ng DNA with 5% sucrose was loaded into 0.65% Megabase agarose (BioRad) in 0.2× Tris–borate buffer and separated at 20 V for 24 h. The gel was stained with SYBR™ Gold Nucleic Acid Gel Stain (ThermoFisher Scientific, USA).

### Human histone octamer preparation

Recombinant human histone proteins were individually expressed, purified, and refolded into human histone octamer, essentially as described^72,73^. Briefly, individual histone proteins were mixed under high salt at a 1.2/1.2/1.0/1.0 (H2A/H2B/H3/H4) ratio, and the assembled octamer was purified by gel filtration chromatography.

### Nucleosome array preparations

The DNA templates and recombinant human histone octamers (HO) were reconstituted into nucleosome arrays by the salt dialysis method described previously^19^, excluding the addition of competitor DNA.

DNA was mixed with HO at three ratios HO:DNA = 0.5, 0.8, and 1.0, calculated based on a ratio of one HO per 197 bp of DNA. The salt concentration was reduced from 2 M NaCl to 10 mM NaCl in continuous dialysis at a 0.65 ml/min flow rate overnight using Masterflex L/S digital Miniflex dual-channel peristaltic pump (Cole-Parmer, US). The final buffer contains 10 mM Tris–HCl (pH 7.5), 0.1 mM EDTA, 1 mM DTT and 10 mM NaCl. The quality of the reconstituted arrays was accessed by electrophoretic mobility shift assay (EMSA); see Fig. S3A.

For the arrays with NRLs shorter than 197 bp (177-38/36, 172-25, 166-28, and 162-27), the reconstitution protocol was similar to the one applied for the λ- and 197 bp NRL arrays (see above). The only difference was that the HO:DNA ratio was close to 1.0 (0.8–1.2). Each array was prepared in three batches of different HO:DNA ratios (e.g., HO: DNA = 0.9, 1.0, and 1.1). Furthermore, two reconstitution runs were performed using DNA templates with long (total length ~ 1000 bp) and short (~ 100 bp) DNA handles. For NRL = 177 bp, the DNA template with long handles contained 36 repeats; the template with short handles had 38 repeats. Long handles of the 177-36 template created space for additional nucleosomes, so the abbreviation ‘177-38’ will be used for both 177-36 and 177-38 arrays.

For all samples, the typical final concentration of the reconstituted arrays was 10–20 ng/µl measured as DNA concentration calculated from solution optical absorption at 259 nm, A_259_.

### Fluorescence microscopy (FM)

λ-DNA (48,502 bp) (New England Biolabs) was used without further purification in FM and negative stain EM experiments. Sample solutions for single molecule observations were prepared by successive mixing of TE buffer (10 mM Tris–HCl, 1 mM EDTA, pH 8) containing 100 mM NaCl, a concentrated solution of λ-DNA or nucleosome array (final DNA concentration of 0.2 µM in phosphates), fluorescent dye YOYO-1 (20 nM) and variable concentrations of MgCl_2_. Solutions were gently mixed after the addition of each new component. The final samples were incubated for 1 h at ambient temperature prior to observations. FM observations were performed using an Eclipse TE2000-U (Nikon, Japan) microscope with a 100× oil-immersion lens. Fluorescent images were recorded and analyzed using an EM-CCD camera and an Argus 10 image processor (Hamamatsu Photonics, Japan). The long-axis length of the individual λ-DNA or nucleosome arrays was measured using ImageJ 1.52a software (NIH)^74^.

### Fluorescent imaging in nanofluidic channels

Nanofluidic devices were fabricated in PDMS elastomer as previously described^27^. The device consisted of an array of 90 μm long, rectangular channels with a cross-section of 50 × 70 nm^2^ (60-nm channel system) or 120 × 130 nm^2^ (125-nm system) and uncertainty in width and depth of ± 5 nm. Before fluorescence imaging, DNA was stained with YOYO-1 (Invitrogen, Carlsbad, CA) at a ratio of one dye molecule for each four base pairs. Then, the stained chromatin was driven into the nanochannels by electrophoresis. The fibers were visualized with a Nikon Eclipse Ti inverted fluorescence microscope equipped with a diode laser, 200 mW/488 nm (Omicron, Germany), filter set, and a 100× oil immersion objective (numerical aperture 1.49). Movie clips at a rate of 2.5 frames/s were recorded with an electron-multiplying charged coupled device camera (Andor iXon X3). The image pixel size of 0.16 × 0.16 μm^2^ was calibrated with the help of a metric ruler.

### Multiplexed magnetic tweezers (MMT)

A detailed account of experimental procedures and data analysis is given in the Supplementary Methods.

A homemade MMT setup and flow cells used in this work were assembled as described before^59,60^. First, a solution of anti-digoxigenin (Merck Millipore, USA; 300 µl at 1 µg/µl) was introduced into the flow cell and incubated for 2 h at room temperature, followed by flushing 1 ml of passivation solution (3.6% Bovine Serum Albumin (BSA) heat shock fraction, pH 7, ≥ 98% (Merck), 0.1% Tween 20 (Merck)) with subsequent storage at 4 °C. A detailed description of the MMT instrument, sample preparation, measurements, and data analysis is given in the “Supplementary Methods” section.

The composition of the fibers varied and was quantified by the total number of nucleosomes in the fiber, *N*_*total,*_ and the number of nucleosomes excluded from stacking and fiber folding *N*_*unfold*_. Values of *N*_*total*_ and *N*_*unfold*_ were obtained by fitting the experimental force-extension curve in the range of moderate (8–15 pN) and low (0.5–2 pN) force worm-like chains that were reduced in contour length by 79 bp × *N*_*total*_ and NRL × *N*_*folded*_ respectively. Next, force-extension curves were fitted with a statistical mechanics model for dynamic unfolding^56^ (see Supplementary Data, Tables S1 and S2), yielding the fiber stiffness (*k*_*fiber*_); free energy (*∆G*_*1*_) associated with nucleosome unstacking, and the first step of DNA unwinding (from 146 to 92 bp); free energy related to the second stage of DNA unwrapping (*∆G*_*2*_; from 92 to 79 bp)^58,59,62^. The number of nucleosomes contributing to the fiber folding is defined as *N*_*folded*_ = *N*_*total*_ − *N*_*unfold*_. Values of *k*_*fiber*_, *∆G*_*1,*_ and *∆G*_*2*_ were automatically fitted after their approximate manual setting. See SD for further explanations.

In this work, we have found that the low-force part of the force-extension curves of the long fibers exhibited abrupt extension jumps that are different from the gradual and reversible unfolding observed for the short arrays. We assign these events to ruptures of contacts between clusters of the folded nucleosomes, separated by stretches of nucleosome-free DNA or non-interacting nucleosomes. Ruptures in the 1 < *F*_*rupture*_ < 15 pN range yielding an extension longer than 68 nm (equivalent to 200 bp of the free DNA) were detected automatically using a Python script, after applying a 15-point median filter to reduce fluctuations and joining events that were separated less than 5 points. The XY-position (dR) change for each rupture was calculated to identify events corresponding to the bead's unsticking from the surface. A threshold of *dR* > 0.1 µm was used to discard such events.

### Negative-stain electron microscopy

λ-array was diluted to a DNA concentration of 5 µg/ml with TEN0.01 buffer and fixed with 0.02% glutaraldehyde on ice for 10 min. 4 µl of the sample was applied on the carbon-coated grid for 1 min and subsequently blotted from the edge of the grid using Whatman Grade 1 filter paper. Immediately, 4 µl of 2% uranyl acetate was applied on the grid and left to stain for another minute. The grids were visualized with a Tecnai T12 electron microscope operating at 120 kV, and micrographs were recorded on Eagle 4 K CCD Camera (FEI) at 30,000–49,000× magnification with a defocus value of − 1.2 μm. Micrographs were analyzed on Fiji and manually counted with the "multi-point" tool. Only arrays that were well defined as single molecules were counted. A total of 17 λ-arrays were counted for each HO:DNA ratio. Due to their large size, the λ-arrays were often entangled with each other, making it difficult to separate the arrays to get large datasets.

### Atomic force microscopy (AFM)

A 5–10 µl droplet of the reconstituted λ-array at 30 ng/µl was spotted on a plasma oxidized silica surface. After 10 min to allow for sample adsorption onto the surface, the specimens were developed by flushing them with 1 ml of ultra-pure water followed by drying in a stream of nitrogen gas. Atomic force imaging was done with a Bruker Dimension Icon microscope (Billerica, MA, USA) at room temperature in the air. Images were acquired in the standard tapping mode with silicon (Si) cantilevers (spring constant of 19–55 N/m) and operated just below their resonance frequency (typically 270–370 kHz). The images were flattened, and the contrast and brightness were adjusted for optimal viewing conditions.

## Supplementary Information

Supplementary Information 1.

Supplementary Video 1.

Supplementary Video 2.

Supplementary Video 3.

Supplementary Video 4.

## Data Availability

The experimental data sets are either included in this submission, the supplemental information, or are available from the authors upon request.

## Abbreviations

AFM: Atomic force microscopy
EM: Electron microscopy
EMSA: Electrophoretic mobility shift assay
FM: Fluorescent microscopy
HO: Histone octamer
MMT: Multiplexed magnetic tweezers
NFR: Nucleosome-free region
NRL: Nucleosome repeat length
TAD: Topologically associating domain
TSS: Transcription start site
WLC: Worm-like chain
