# Supplementary material for: Reconstituted TAD-size chromatin fibers feature heterogeneous nucleosome clusters
Source: Sci Rep. 2022 Sep 16;12:15558. doi: 10.1038/s41598-022-19471-3 (PMC9481575; doi:10.1038/s41598-022-19471-3)
Supplement: Supplementary file 1 — Supplementary Information 1. [file 41598_2022_19471_MOESM1_ESM.pdf]

Supplementary Information

Reconstituted TAD-size chromatin fibers feature heterogeneous nucleosome clusters

Nikolay Korolev, Anatoly Zinchenko, Aghil Soman, Qinming Chen, Sook Yi Wong, Nikolay V. Berezhnoy, Rajib Basak, Johan R. C. van der Maarel, John van Noort, and Lars Nordenskiöld

Supplementary Methods

Preparation of DNA templates with '601'nucleosome positioning sequence for the MMT measurements
The 197-601-15 DNA (15 repeats of the Widom's '601' high affinity nucleosome positioning sequence with 197 bp NRL), as well as the other '601' DNA array constructs, are prepared with a controlled NRL and number of repeats, were initially constructed in a pUC18 vector following published protocols[1-3]. The DNA constructs 197-15, 177-38, and 166-28 were kind gifts from Daniela Rhodes, while the others were prepared following the published protocols[1-3]. The plasmid with 197-15 DNA was transformed into TOP10 (Invitrogen) competent cell for amplification. The amplified plasmid was digested with EcoRI, XbaI, HaeII, and DdeI (New England Biolabs), followed by PEG6000 fractionation. The 197-15 DNA fragment was further purified by HiPrep 26/60 Sephacryl S-500 HR column (GE Healthcare). To generate long DNA handles for tweezer experiments, PCR was conducted with the pCU18 vector and two oligo primers containing digoxigenin and biotin labels at the 5'- and 3'-ends, respectively. This generated a 1-kb long DNA fragment. The PCR product was further digested with EcoRI and XbaI to get the ~412 bp digoxigenin-labeled handle with EcoRI overhang and the ~596 bp length biotin-labelled handle two with XbaI overhang. To achieve torsionally free DNA templates for the MMT measurement, 197-15 DNA and the handles were ligated to the EcoRI, and XbaI overhangs in a T4 ligase reaction (Supplementary **Fig. S1C**). The one-step ligation method of the 197-15 DNA labeling resulted in multiple products (see Supplementary **Fig. S2B**). Among these products, only three have different (biotin and digoxigenin) labels on their termini and are applicable for the magnetic tweezers measurements; doubly labeled 197-15, 197-45, and 197-75 DNA templates (more details are given in the legend to Supplementary **Fig. S2**).

In addition to studying nucleosome arrays reconstituted on the λ-DNA and '601'-197 bp NRL DNA templates, we examined several nucleosome arrays generated from the '601' positioning DNAs with NRLs ranging from 177 to 162 bp, namely 177-38/36, 172-25, 166-28, and 162-27 (the first number indicates the NRL, the second one the number of NRL repeats). For the MMT measurements, torsionally free constructs of these DNA templates were obtained in two preparations using short (~25 bp) and long (~500 bp) handles. The preparation of '601' DNAs with long handles is the same as for 197-15 DNA. To prepare DNA with short handles, a purified plasmid containing 601 DNA was digested with EcoRI and HindIII to create non-complementary overhangs at both ends for short handle ligation. The short handles were achieved by annealing the oligo-Dig label and oligo-Biotin label with complementary oligomers. These asymmetric short linkers of ~25 bp were ligated to 601 DNA fragments with the EcoRI and HindIII overhangs in a T4 ligase reaction (Supplementary **Fig. S1C**).

Human DNA. Expi 293 cells were provided by the Protein Production Platform at the School of Biological Science, NTU. The nuclei were prepared following the Abcam nuclei extraction protocol with minor modifications. The cells were pelleted and resuspended in chilled (4°C) buffer containing 20 mM HEPES (pH 7.5), 1.5 mM $MgCl_2$, 10 mM KCl, 0.5 mM DTT, 1 mM PMSF and 0.05% NP40. The resuspended cells were homogenized using a Dounce homogenizer, followed by centrifugation at 1,000 g for 10 minutes at 4°C. The nuclei were resuspended in MNnase buffer to 2 mg/ml DNA concentration and digested with 50 U of MNase (New England Biolabs, Pte. Ltd, Singapore) per ml of nuclei for 1-3 minutes. The reaction was stopped by adding EDTA to 10 mM final concentration. The digested nuclei was centrifuged at 20,000g for 5 min at 4°C followed by extraction with buffer (20 mM TRIS

(pH 7.5), 1 mM EDTA, 1 mM DTT) containing an increasing amount of NaCl (80, 160, 300 mM). This wash removed approximately oligonucleosomes up to 4 nucleosomes, and longer chromatin fragments were contained in the pellet. The native chromatin contained in the pellet, due to their enhanced protection compared to the oligonucleosomes removed in elevated salt washes, is inferred to contain native DNA occupied by nucleosomes. The pellet of chromatin fragments was resuspended in 20 mM TRIS, 1 mM DTT, and 0.5% SDS by pipetting and treated with proteinase K (50 µl/ml) (New England Biolabs, Pte. Ltd) at 37°C for 2 hours. The sample was extracted with Phenol-Chloroform-Isoamyl alcohol (25:24:1) three times, followed by two extractions with Chloroform: Isoamyl alcohol (24:1). The DNA was precipitated by the addition of 3× volume of ethanol. The pellet was washed with 70% ethanol twice, resuspended in 10 Tris (pH 8.0), EDTA 0.1 mM, and used for downstream applications.

Multiplexed Magnetic Tweezers (MMT) measurements

MMT design. A homemade MMT setup was assembled following the inverted microscope design developed in the laboratory of Chromatin Dynamics of Leiden Institute of Physics, Huygens-Kamerlingh Onnes Laboratory, Leiden University[4,5]. The dynamic force spectroscopy measurements were done on a sample illuminated by a 100 µW LED collimator (IMM Photonics GmbH, Germany) placed in a custom-built flow cell[4,5] positioned on an XY stage (OWIS GmbH, Germany), while the magnet changes position and the response of the magnetic beads was monitored using NIKON CFI Plan Apochromat Lambda NA 0.75, 20× objective (Nikon Corporation, Japan), infinity-corrected tube lens ITL200 (Thorlabs, USA) and 25 Mpix Vieworks monochrome camera (Vision Systems Technology, USA). The magnet probe was made of two closely aligned antiparallel NeFeB N50 magnets (Supermagnete, Webcraft GmbH, Germany). The magnet and the objective were positioned by M-126 translational stages (Physik Instrumente GmbH, Germany) and, together with the stepper motors (Intelligent Motion Systems, USA), connected to the XY stage, were controlled by a 6-axis stepper motor driver (Trinamic, Germany). The Frame Grabber Camera Link PCIe-1433 (National Instruments, USA) was installed in an 8-core Intel Xeon 3.2 GHz processor (Intel Corp., USA) with 32 GB ECC memory.

Flow cells used in this work were designed in the Chromatin Dynamics Laboratory of Leiden Institute of Physics[4,5]. The flow cell consists of an aluminum bottom mold and a polycarbonate top mold. The metal mold has channels with threads to accommodate FEP tubing (outer diameter 1/16″) held using the super flangeless ferrule system (IDEX Health and Science LLC, USA). The top mold has holes for electric wires (outer diameter 0.02″ matching the inner diameter of the tubings) that are used to block tubing during the injection step and an injection port. The Sylgard 184 Silicone Elastomer kit (Dow, USA) liquid was injected into assembled molds held by M4 bolts between the cover glass 24×40 mm, #1 thickness (Gerhard *Menzel,* Glasbearbeitungswe, Germany) positioned in the metal mould, and the acrylic top mould. A piece of Dymo tape shaped like a channel was stuck to the inner side of the acrylic top to guide subsequent poly(dimethylsiloxane) (PDMS) removal using a scalpel to form a channel connecting the outlets of two tubings. The dimensions of the resulting channel were about 42 × 10 × 1 mm. Cover glass 24×60 mm, #1 thickness (Gerhard *Menzel,* Glasbearbeitungswe, Germany) covered by 0.1 % collodion solution (Merck Millipore, USA) was applied to the top of the aluminum mould to seal the flow cell. A watertight flow cell allows the application of solutions by pump or manually using a syringe. 300 µL solution of anti-digoxigenin (Merck Millipore, USA) at 1 µg/µL was introduced into the flow cell and incubated for 2 hours at room temperature, followed by flushing 1 mL of

passivation solution (3.6 % Bovine Serum Albumin (BSA) heat shock fraction, pH 7, ≥ 98% (Merck), 0.1% Tween 20 (Merck)) with subsequent storage at 4°C.

Sample preparation and measurements. Nucleosome array fibers were reconstituted using the salt dialysis method. Three preparations of the arrays were made, testing three different HO:DNA ratios in each of the reconstitutions: HO:DNA ratios were 0. 5, 0.8, and 1.0 using NRL value 200 bp for calculation of the HO amount. The quality of reconstitution was checked on 0.7% agarose EMSA using freshly reconstituted nucleosome arrays (Supplementary **Fig. S1**)

The flow cell was washed from passivation solution with 1 mL of measurement buffer, MB (100 mM KCl, 2 mM $MgCl_2$, 10 mM NaN_3, 10 mM HEPES pH 7.5, 0.1% TWEEN 20, 0.2% BSA). To attach the biotin-labeled end of DNA to streptavidin-coated beads, 2 µL of vortexed 10 mg/mL 2.8 µm paramagnetic beads (Dynabeads M-280 Streptavidin, Thermo Fisher Scientific Baltics UAB, Norway) was added to 0.1-1.0 pg/µL solution of nucleosome arrays in 500 µL of MB followed by incubation for 10 min with gentle mixing. Then, 500 µL of MB with the beads and the arrays attached were pumped into the flow cell by a syringe pump (New Era Pump Systems, USA) at 200 µL/min. The cell was incubated for 10 min at room temperature to bind the digoxygenin-labeled end of DNA to the cover glass surface. The unbound beads were removed by flushing the flow cell with 500 µL of MB at 200 µL/min.

Data collection was performed using procedures and scripts in the LabVIEW environment (National Instruments, USA) developed in the Chromatin Dynamics Laboratory of Leiden Institute of Physics and described in detail in[4-7]. Before nucleosome array measurements, force calibration for the given MMT setup and batch of magnetic beads was performed as described[5]. It was found that both single and double exponent fittings gave coincided force–magnet position calibrations in the range 0.1 – 60 pN. To determine the force (F) acting on the bead, a single-exponent fitting equation was used:

$$F\ (\mathrm{pN}) = 0.02 + 68\cdot\exp(-shift/0.96396) \quad (1)$$

where *shift* is a magnet position relative to the flow cell surface in millimeters.

After manually adjusting the objective position, magnetic beads were automatically picked up and, if necessary, manually filtered, removing fault hits, double and stuck beads. Typically, 10 – 0 – 10 mm magnet shift of one or two 80-120 sec cycles of the fibers' stretch – relief was recorded. Not more than 3-4 measurements were carried out in each flowing cell, ensuring that the magnets' displacement was more than 5 mm relative to a preceding field of view[4]; that is, the beads were not subjected to force exceeding 1 pN. Positions of the beads in three dimensions were monitored, applying recent 2D Fast Fourier Transforms algorithms to compute cross-correlations with computer-generated reference images[6].

Data analysis. A statistical mechanics model developed on the LabView platform in the Chromatin Dynamics Laboratory of Leiden Institute of Physics was used to interpret the force-extension data of the nucleosome array stretching[4,5,7,8]. The model describes the dependence of total extension (z_{tot}) of the nucleosome fiber along the direction of the applied force, F, (Figure 1 of the main text) as a sum of five terms related to:

0). stretching of the free DNA;

1). extension caused by deformation of the condensed chromatin fiber;

2). fiber extension to a beads-on-a-string chain associated with the nucleosome-nucleosome unstacking and simultaneous partial unwinding of an outer-turn DNA from the HO;

3). deformation of the individual nucleosomes with further DNA unwinding and disruption of the histone octamer that might be accompanied by dissociation of the H2A/H2B dimers from the $(H3/H4)_2$ tetramer;

4). step-wise abrupt ruptures of the inner DNA turn in each of the nucleosomes with complete stretching of the DNA and largely irreversible histone loss.

Equations applied in the model[4,7,8] are described below and summarized in Supplementary **Table S1**.

At all stages of tether stretching, chromatin fiber contains DNA that is not bound to the histone octamer. The mechanical response of this DNA with contour length L to the applied force F is described in terms of free energy $G_{DNA}(F, L)$ and extension $z_{DNA}(F, L)$ by a worm-like chain model:

$$G_{DNA}(F,L) = -L\left[F - \sqrt{\frac{Fk_BT}{A}} + \frac{F^2}{2S}\right] + Fz_{DNA} \quad (2a)$$

$$z_{DNA}(F,L) = L\left[1 + \frac{1}{2}\sqrt{\frac{k_BT}{FA}} + \frac{F}{S}\right] \quad (2b)$$

where $G_{DNA}(F, L)$ is expressed in k_BT units (k_B is Boltzmann constant, T temperature); A and S are the persistence length and the DNA stretching modulus.

Total free energy $G_{tot}(F)$ and extension $z_{tot}(F)$ of the chromatin fiber with N nucleosomes represent contributions from each nucleosome and free DNA. Free DNA includes the DNA of the tether handles plus DNA of the nucleosomes that have been either not reconstituted or lost before the fiber stretching.

$$G_{tot}(F) = \sum_{i=1}^{N} G_i(F) + G_{DNA}(F, L_{free}) \quad (3a)$$

$$z_{tot}(F) = \sum_{i=1}^{N} z_i(F) + z_{DNA}(F, L_{free}) \quad (3b)$$

For the cases when nucleosomes are indistinguishable, and there might exist many fiber conformations with the same energy and extension, the model includes a degeneracy factor $D(state)$:

$$D(state) = \prod_{i<j} \binom{n_i + n_j}{n_i} \quad (4)$$

where n_i and n_j are respectively the numbers of the nucleosomes in states i and j. With the inclusion of the degeneracy, averaged extension of the fiber in dependence of applied force can be calculated from standard statistical mechanics Boltzmann equation:

$$\langle z_{tot}(F)\rangle = \frac{\sum_{states} z_{tot}(F) \cdot D(state) \cdot e^{-(G_{tot}(F) - Fz_{tot})/k_BT)}}{\sum_{states} D(state) \cdot e^{-(G_{tot}(F) - Fz_{tot})/k_BT)}} \quad (5)$$

Equation (5) was fitted to the experimental force-extension curves, with several parameters used in the model fixed. These fixed values are listed in **Table S2**, and they are either known numbers specific for a given nucleosome array (contour length of the template DNA, nucleosome repeat length (NRL)), well-established properties of the free DNA (persistence length, stiffness) or parameters verified earlier in other studies including tweezers measurements of the nucleosome arrays[4,9,10].

Table S1. Equations describing free energy and extension terms used in the statistical mechanics model of the nucleosome array stretching.

Nucleosome wrapping state	Free energy, $G_i(F,L)$	Extension, $z_i(F,L)$
Extension of chromatin fiber	$G_{fiber}(F) = \frac{F^2}{2k_{fiber}}$	$z_{fiber}(F) = \frac{F}{k_{fiber}} + z_0$
Unstacking and partial DNA unwrapping	$G_{partially\ wrapped}(F) = G_{DNA}(F, L_{partially\ wrapped}) + \Delta G_1$	$z_{partially\ wrapped}(F) = z_{DNA}(F, L_{partially\ wrapped}$
DNA unwrapping to a single turn	$G_{singly\ wrapped}(F) = G_{DNA}(F, L_{singly\ wrapped}) + \Delta G_1 + \Delta G_2$	$z_{singly\ wrapped}(F) = z_{DNA}(F, L_{singly\ wrapped})$
DNA fully unwrapped	$G_{fully\ unwrapped}(F) = G_{DNA}(F, L_{fully\ unwrapped}) + \Delta G_1 + \Delta G_2 + \Delta G_3$	$z_{fully\ unwrapped}(F) = z_{DNA}(F, L_{fully\ unwrapped})$

Here, k_{fiber} is the stiffness of the fiber; z_0 nucleosome line density; $L_{partially\ wrapped}$ and $L_{singly\ wrapped}$ are respectively lengths of the DNA released during transformations of the NCP from folded fiber to a bead-on-a-string chain and stretched nucleosome states; $\Delta G_1, \Delta G_2, \Delta G_3$ are respective changes in free energy associated with each step of nucleosome unwinding.

Experimental force-extension curves were fitted to the model as described in detail in[4]. First, experimental data were manually shifted to correspond to the length of fully stretched DNA, modifying z-coordinate offset and drift. Followed fitting uses fixed and adjusted parameters. The fixed parameters are either known, or expected numbers specific for a given nucleosome array (contour length of the template DNA, nucleosome repeat length, NRL), well-established properties of the free DNA (persistence length, stretch modulus), or parameters verified earlier in other studies including tweezers measurements of the nucleosome arrays[4,9,10]. Fixed parameters for the DNA and the nucleosome fiber are listed in Supplementary **Table S2**.

The following values were explicitly fitted for each stretching curve:

i) The total number of nucleosomes in the fiber (N_{total})

ii) The number of nucleosomes not included in the folded fiber (N_{unfold}). These might be nucleosomes lacking one or two H2A/H2B dimers (hexasomes and tetrasomes) or complete nucleosomes outside the folded fiber domain and not participating in nucleosome – nucleosome interactions.

iii) The fiber stiffness (k_{fiber}).

iv) The free energy (ΔG_1) associated with the combined event of nucleosome unstacking and partial DNA unwrapping.

vi) Free energy related to the second stage of DNA unwrapping up to a single turn of DNA (ΔG_2). Based on the data of earlier studies[4,8,10], the number of the unwrapped DNA base pairs was fixed at 13 bp (corresponding to the decrease of the DNA attached to the HO from 92 to 79 bp).

The values of N_{total} and N_{unfold} were set manually by adjusting the fitting curve to the experimental points. N_{total} was determined from the high-force part (> 8 pN) of the stretching curve corresponding to the ruptures of the last 79 bp DNA from the histone core; N_{unfold} was fitted from experimental data at the low-force range (0.5 – 2 pN). In the model, these unfolded nucleosomes undergo only the last-turn rupture event. The number of nucleosomes

contributing to the fiber folding is defined as $N_{folded} = N_{total} - N_{unfold}$. It is not possible[9] to resolve the differences between the complete and incomplete (hexasomes and tetrasomes) nucleosomes in the last rupture event, and it is assumed that both the populations are characterized by the same last step in the unwrapping pathway. The k_{fiber}, ΔG_1, and ΔG_2 values were automatically fitted after initial guesses based on previous data. All traces analyzed in this work produced the best fitting to the experimental data when the degeneracy parameter was set equal to one.

For the arrays reconstituted on the MNase-digested genomic DNA (native-DNA arrays), the fitting procedure included adjusting the DNA length and setting the shift and drift of the magnetic bead.

Table S2. Fixed parameters used to fit the experimental data to the statistical mechanics model of the λ-DNA arrays' stretching.

Parameter (units)	Value
DNA contour length (bp)	48,548
Nucleosome repeat length, NRL (bp)	197
DNA persistence length (nm)	50
DNA stiffness (pN)*	900-1500
Folded fiber length, z_0 (nm per nucleosome)	1.5
Partially wrapped length (bp per nucleosome)	55
Singly wrapped length (bp per nucleosome)	79
Free energy of single wrap rupture, ΔG_3 (k_BT)	90

*For each experimental stretching trajectory, DNA stiffness modulus was set within the range reported in the literature (1000 – 1500 pN) by adjusting the slope of the force-extension fitting curve to experimental data at high force (above 30-40 pN).

Due to a large number of nucleosomes in the reconstituted arrays, the last stage of the stretching, largely irreversible ruptures of 75-80 bp DNA, was impossible to resolve with one-rupture precision.

Supplementary Figures and Tables

Supplementary Information includes 4 movies:

Supplementary Movie 1: dynamics of λ-chromatin reconstituted at HO:DNA ratio 0.5 in the 60 nm channel, filename: "Supplementary Movie 1 lambda-array at HO to DNA ratio 0.5 in 60 nm channel.mp4"

Supplementary Movie 2: dynamics of λ-chromatin reconstituted at HO:DNA ratio 1.0 in the 60 nm channel, filename: "Supplementary Movie 2 lambda-array at HO to DNA ratio 1.0 in 60 nm channel.mp4"

Supplementary Movie 3: dynamics of λ-chromatin reconstituted at HO:DNA ratio 0.5 in the 100 nm channel, filename: "Supplementary Movie 3 lambda-array at HO to DNA ratio 0.5 in 125 nm channel.mp4"

Supplementary Movie 4: dynamics of λ-chromatin reconstituted at HO:DNA ratio 1.0 in the 100 nm channel, filename: "Supplementary Movie 4 lambda-array at HO to DNA ratio 1.0 in 125 nm channel.mp4"

The scale bar denotes 2 micrometers.

Supplementary Figure S1

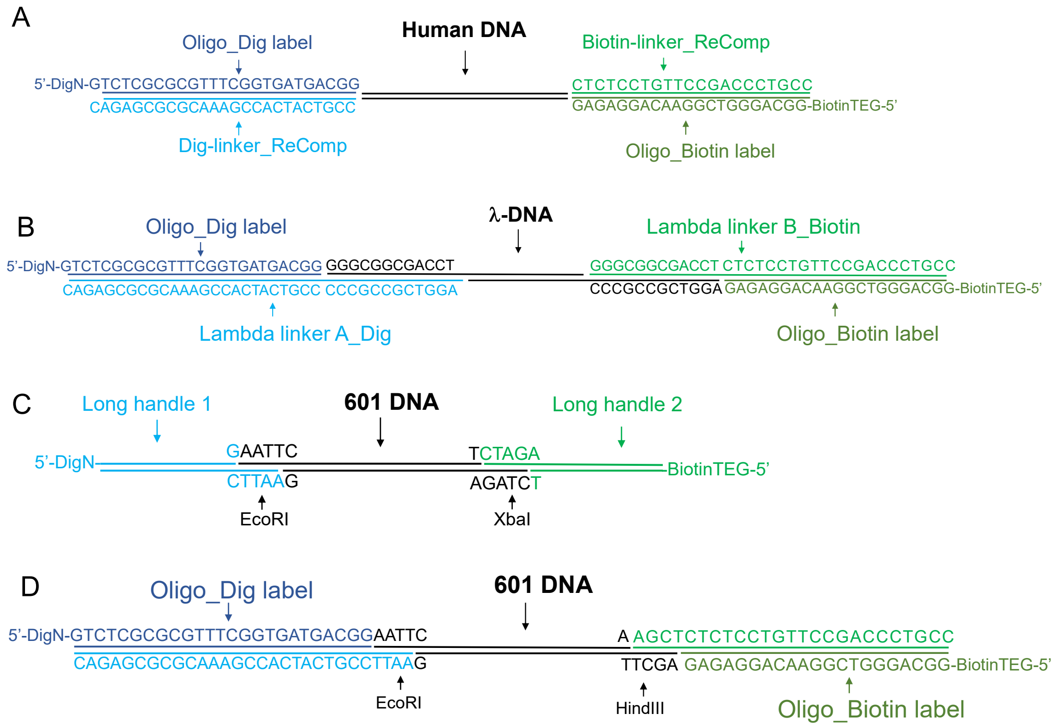

Figure S1. Preparation of the labeled DNA. Scheme of the DNA labeling for magnetic tweezers measurements. (**A**). Products of the MNase digestion of the genomic DNA. (**B**). λ-DNA. (**C**). Labeling of the '601' DNA using long handles. DNA labeling resulted in the addition of the 1,005 bp to the DNA template. (**D**). Labeling the '601' DNA using short handles leads to lengthening the DNA template by 102 bp.

Supplementary Figure S2

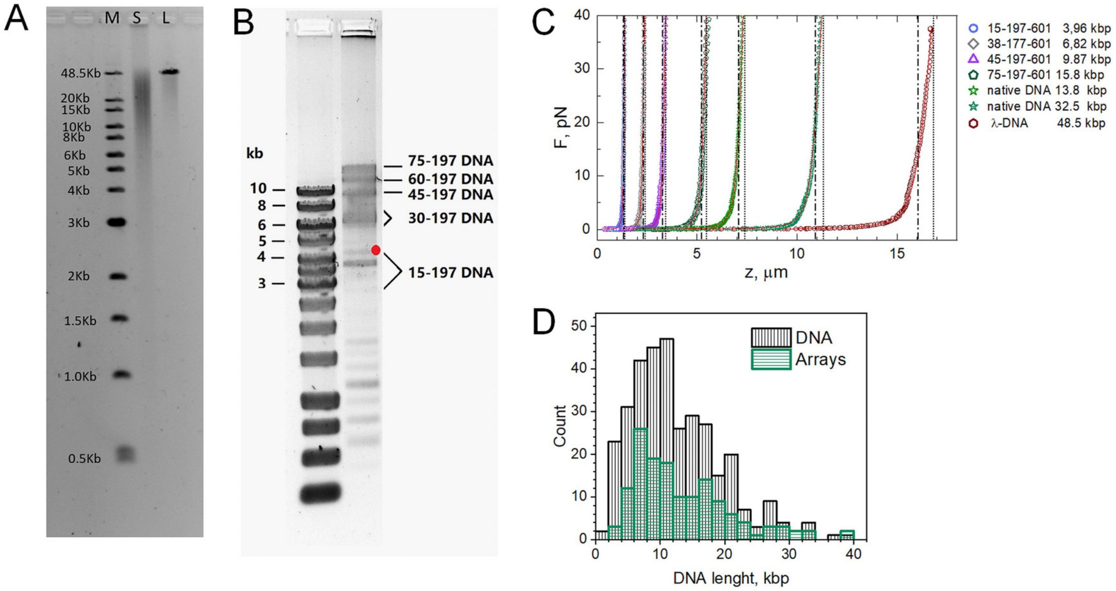

Figure S2. Characterization of the DNA size and labeling. (**A**). Pulse-field gel electrophoresis analysis of the MNase-digested genomic DNA (line S) and λ-DNA (line L, size 48.5 kbp). Sizes of the genomic DNA fragments range from ~5 kb to 40Kb. Line M is a DNA-size marker. (**B**). Agarose gel electrophoresis of the labeled DNA sample with 15 repeats of the 197 bp NRL '601' sequence. Ligation of the single 197-15 DNA gives four products as four bands in the gel (197-15, 197-15 + long handle 1 or long handle 2, 197-15 + both handles). Only the sample in the band of 197-15 + 2 handles (marked with a red circle) can be measured by the magnetic tweezers method. Also, the 197-15 DNA can self-ligate during the ligation process, leading to the formation of 197-30, 197-45, 197-60, and 197-75 products observed in the gel. Theoretically, there should be four bands (197, 197 + handle 1 or handle 2, 197 + both handles) for each self-ligated product, the same as for 197-15 DNA. However, those bands cannot be resolved in the gel due to their large size. Since only the DNA samples containing two different labels are suitable for the MMT method, the 197-30 and 197-60 DNA products are not measurable because they carry the same labels at their ends. (**C**). Sample curves of stretching λ-DNA, labeled genomic DNA, and '601' positioning DNA. Points are experimental data; smooth curves are the data fitting to the WLC model. Dashed and dotted vertical lines show the contour lengths of DNA and stretched DNA, respectively, at 40 pN of the applied force calculated using expected or fitted lengths of the labeled DNA samples. (**D**). Distribution of the MNase-digested genomic DNA lengths measured by the MT method for the DNA (black) and reconstituted nucleosome arrays (green). OriginPro software[11] (www.originlab.com) was used to create the graphs in panels **C** and **D**.

Supplementary Figure S3

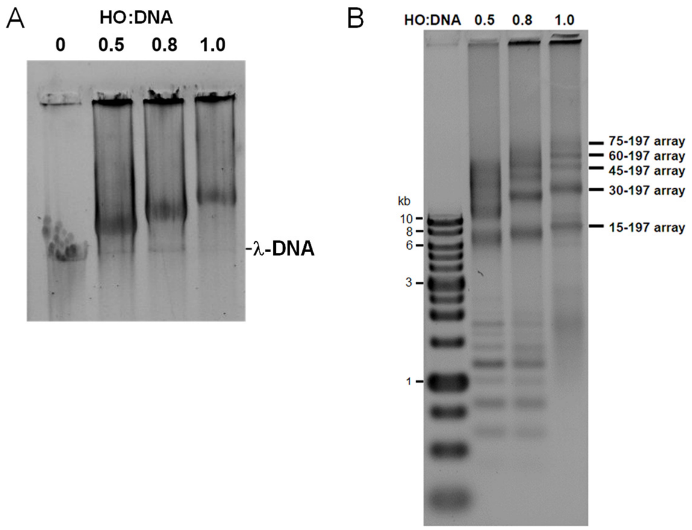

Figure S3. Preparation of the nucleosome arrays. (A). Agarose gel electrophoresis of the λ-DNA (HO:DNA = 0) and the reconstituted λ-DNA arrays with HO:DNA ratios 0.5, 0.8 and 1.0. (**B**). Agarose gel electrophoresis of the nucleosome arrays containing 197 bp '601' positioning sequence with HO:DNA ratios 0.5, 0.8, and 1.0.

Supplementary Figure S4

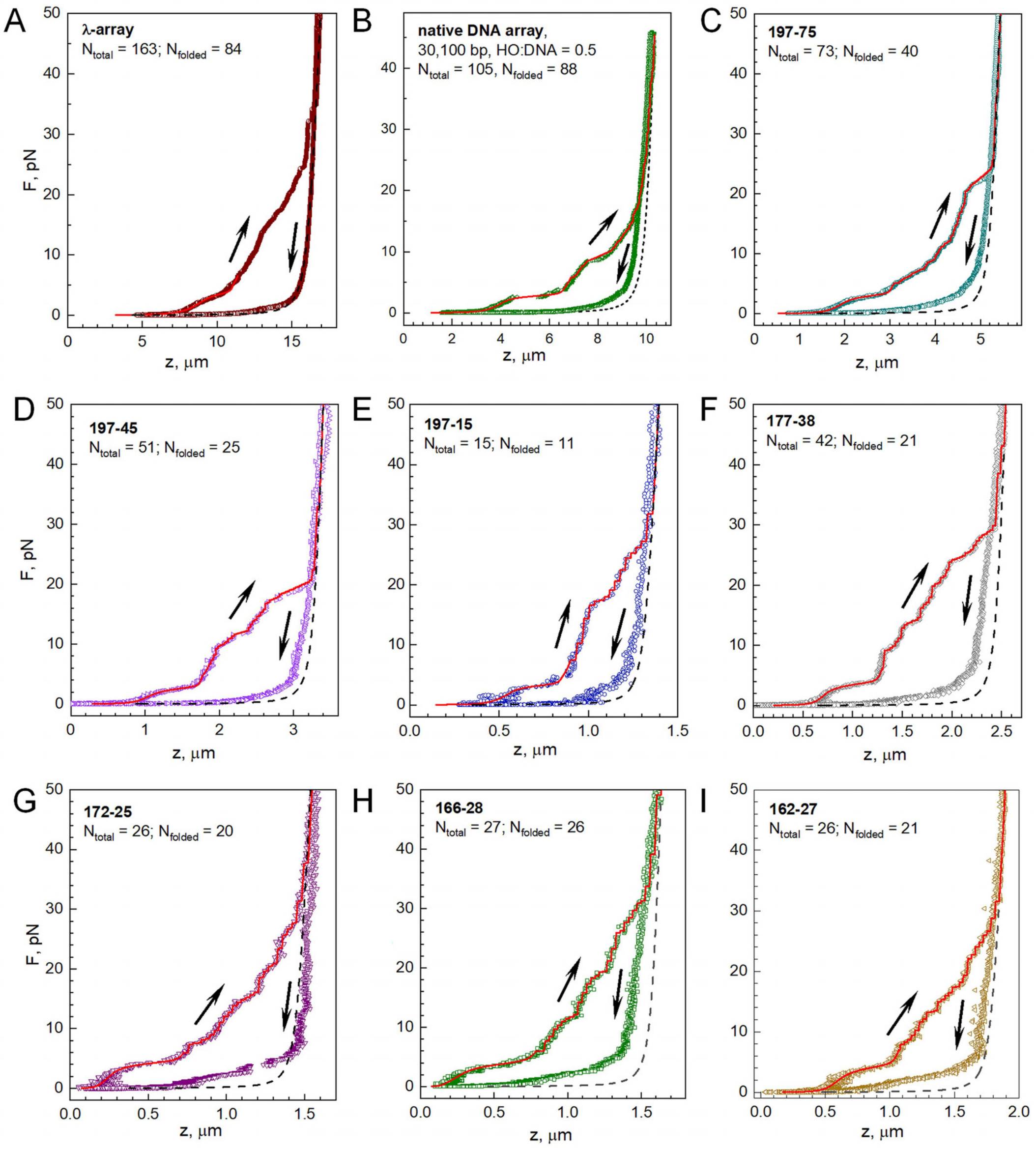

Figure S4. Examples of the stretch-relief curves of the nucleosome arrays reconstituted on the DNA from different sources, with variable NRL, number of nucleosome repeats and DNA length. The number of the repeats and NRL are indicated in the graphs, and the numbers of total (N_{total}) and folded (N_{folded}) nucleosomes determined by fitting the experimental data to the statistical mechanics model. Red solid lines are the fitting curves. Dashed lines display force-extension curves calculated for the bare DNA using the WLC model. All data except the native DNA array (panel B; HO:DNA) were obtained for the arrays with a saturated HO:DNA ratio of 1.0. In each graph, arrows indicate stretching (arrowhead up) and relief (arrowhead down) parts of the force-extension data. OriginPro software[11] (www.originlab.com) was used to create the graphs.

Supplementary Figure S5

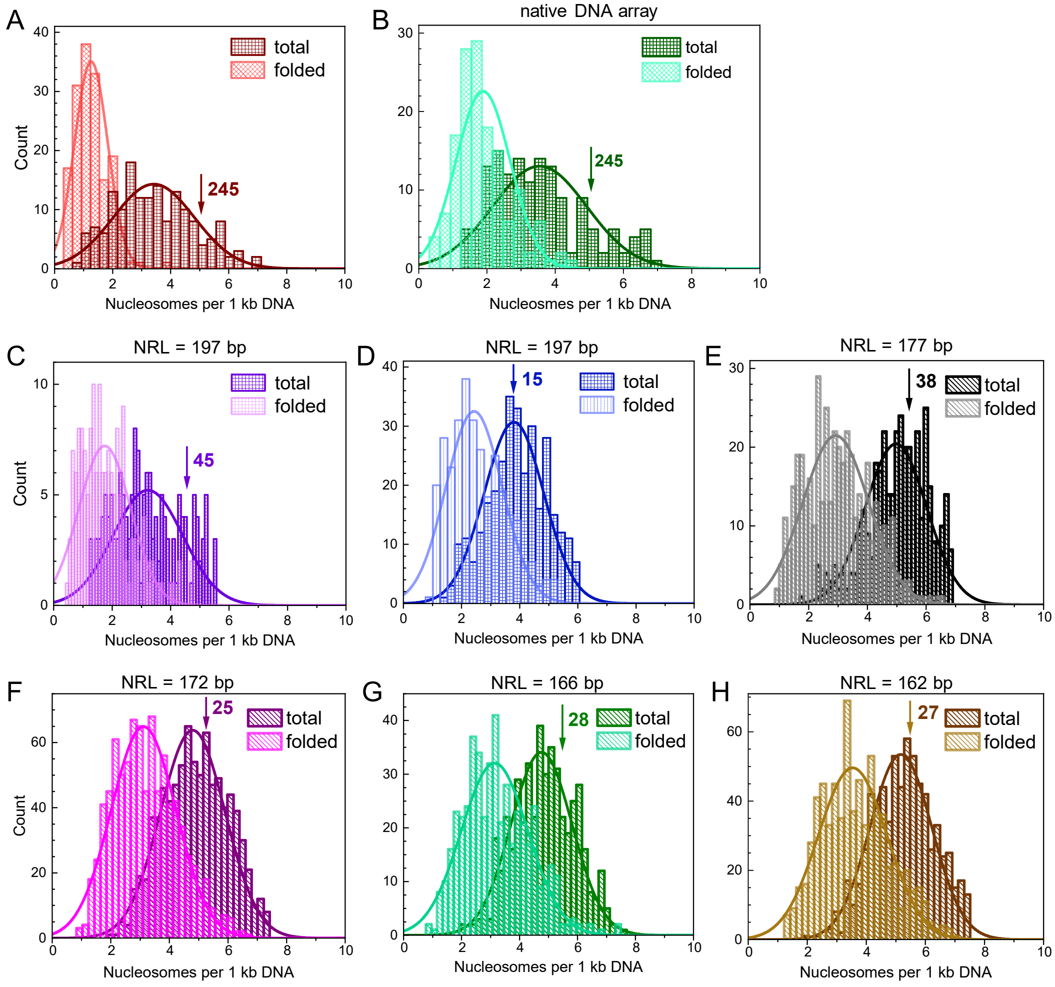

Figure S5. Distributions of the total number of nucleosomes (N_{total}) and the number of the nucleosomes in the folded state (N_{folded}) in the arrays reconstituted on the λ-DNA (**A**), MNase-digested genomic DNA (**B**), and on the '601' DNA positioning sequences (**C-H**). Nucleosome repeat length of the '601'-DNA based arrays is shown on the top of the graphs; the number of the nucleosome repeats is indicated by an arrow and a number in each panel; for the λ-array (A) number of nucleosomes (245) is estimated assuming NRL = 197 bp. In each graph, curves show the data fitting to normal distribution. The number of nucleosomes can be larger than the number of the '601' DNA repeats is explained by the formation of the nucleosomes on the flanking DNA stretches (about 1 kbp). The arrays with NRLs from 177 to 162 bp (panels **E-H**) were reconstituted at the HO:DNA ratios close to saturation (0.9 – 1.1). For the λ-array (**A**) and for the arrays with NRL = 197 bp (**C**, 197-45; **D**, 197-15), the distribution was built using all recorded data obtained for three HO:DNA ratios (0.5, 0.8, and 1.0); for the native DNA arrays (**B**) the HO:DNA ratios were 0.5, 0.8, and 1.0. This resulted in a broadening of the distributions and a higher proportion of the nucleosomes with lower N_{total} and N_{folded} numbers. For the arrays reconstituted on the MNase-digested genomic DNA (**B**) with the DNA lengths varied in a broad range, the observed values N_{total} and N_{folded} were projected to the imaginary DNA length equal to that of the λ-DNA: $N_{projected} = N_{observed} \cdot (48{,}548\ \text{bp}/L_{DNA,observed})$. OriginPro software[11] (www.originlab.com) was used to create the graphs.

Supplementary Figure S6

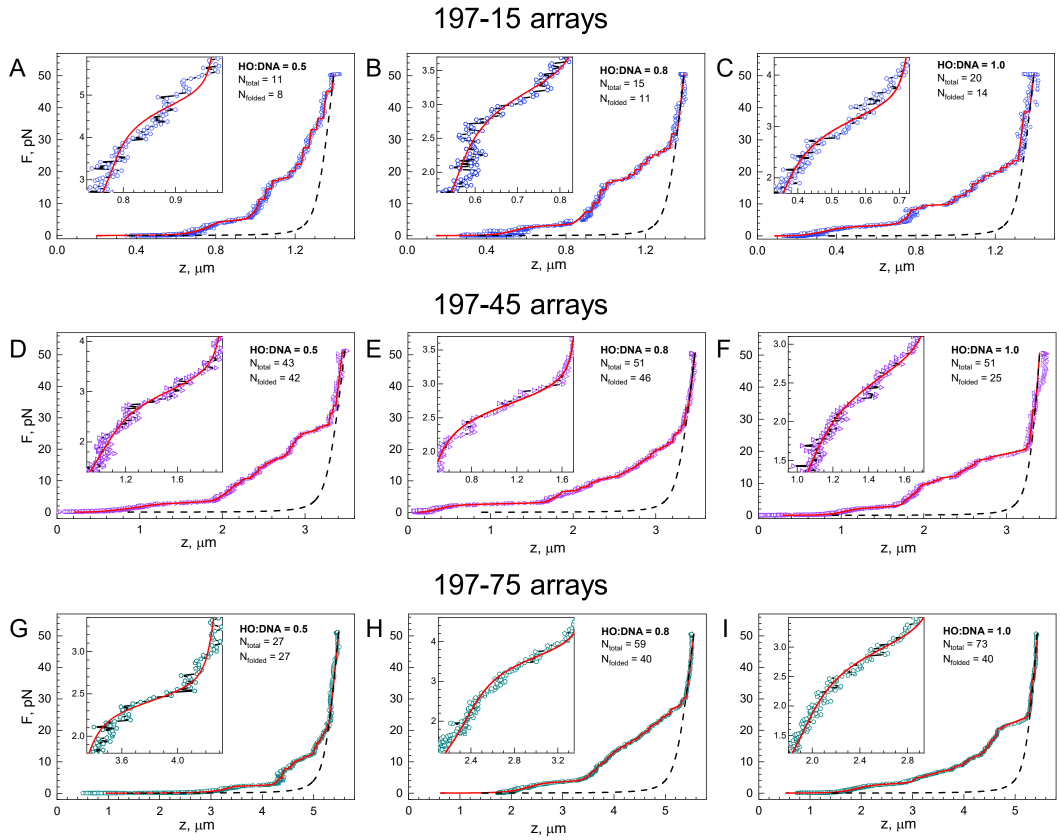

Figure S6. Examples of the force-extension curves of the nucleosome arrays with 197 bp NRL with 15 (**A-C**), 45 (**D-F**), and 75 (**G-I**) nucleosome repeats with the various stoichiometry of the histone octamer – 197 bp DNA repeat: 0.5 (left column, panels **A, D, G**); 0.8 (central column, panels **B, E, H**); and 1.0 (right column, panels **C, F, I**). Inserts in each graph display low-force regions corresponding to the stretching of the folded nucleosome arrays. Points connected by the black lines are experiential data, and smooth red curves are the model fitting. The numbers of total (N_{total}) and folded (N_{folded}) nucleosomes determined by fitting the experimental data to the statistical mechanics model for each measurement are indicated in the panels. Dashed lines display force-extension curves calculated using the WLC mode and the DNA contour length for the bare DNA. OriginPro software[11] (www.originlab.com) was used to create the graphs.

Supplementary Figure S7

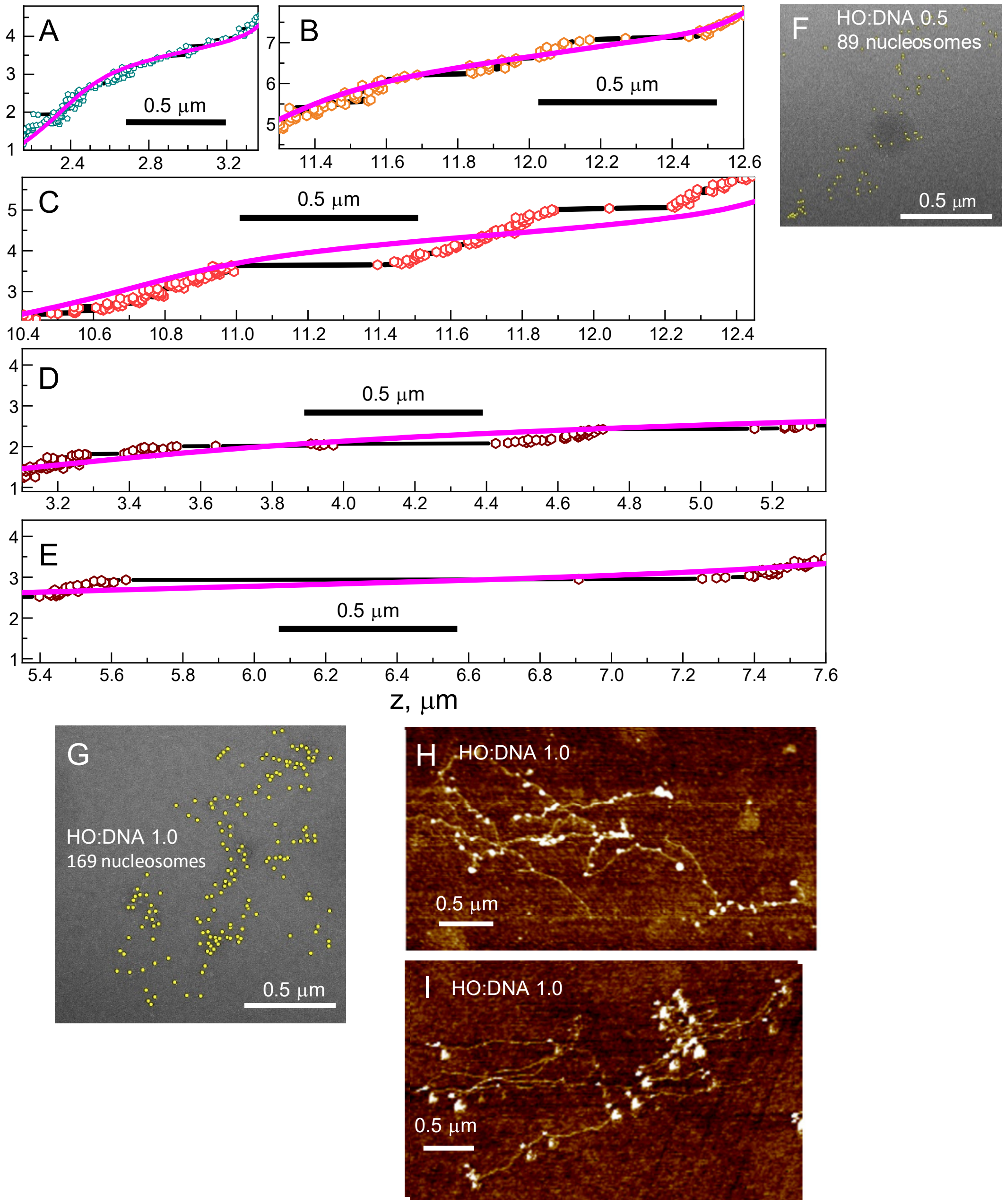

Figure S7. λ-arrays form heterogeneous nucleosome clusters. Comparison of the data of single-molecule array stretching with EM and AFM imaging. (**A-E**) Sample curves of the array stretching in the region on fiber unfolding. (**A**) Model array reconstituted on the 197-75 DNA; λ-arrays with HO:DNA ratios 0.5 (**B**), 0.8 (**C**) and 1.0 (**D, E**). Points connected by the black lines are experiential data, and smooth magenta curves are statistical mechanics model fitting. (**F-I**) Images of the λ-arrays obtained for the HO:DNA 0.5 (**F**) and 1.0 (**G-I**) by the negative staining EM (**F, G**) and the AFM (**H, I**) methods. In each panel, the horizontal bar corresponds to 0.5 μm. OriginPro software[11] (www.originlab.com) was used to create the graphs in panels **A-E**.

Supplementary Figure S8

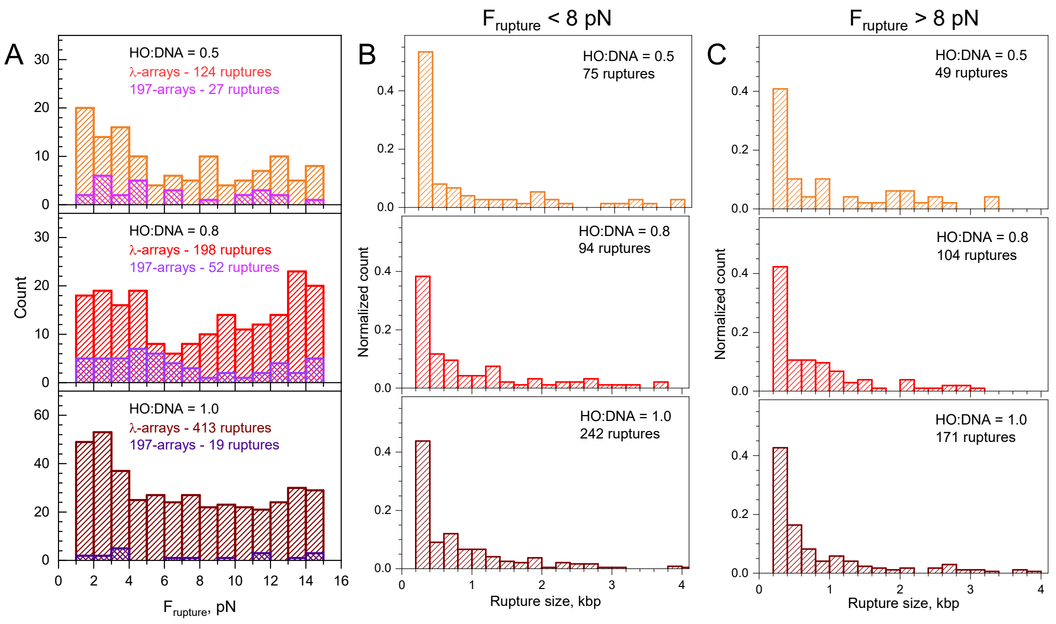

Figure S8. Analysis of the cluster-cluster ruptures in the λ- and 197- arrays. (**A**). Distribution of rupture force ($F_{rupture}$) observed for the λ-arrays with HO:DNA ratios 0.5 (top, blue bars), 0.8 (middle, red bars), and 1.0 (bottom, dark red bars) in comparison with the data obtained for the 197-45 arrays with similar HO:DNA ratios (black bars). The total number of ruptures recorded for each array type is indicated in the graphs. (**B**) and (**C**). Normalized distribution of the cluster-cluster distance determined for the λ-arrays for $F_{rupture}$ < 8 pN (**B**) and $F_{rupture}$ > 8 pN (**C**). HO:DNA ratios and numbers of the observed ruptures are indicated on the graphs. The distance is expressed in kbp DNA. OriginPro software[11] (www.originlab.com) was used to create the graphs.

Supplementary Figure S9

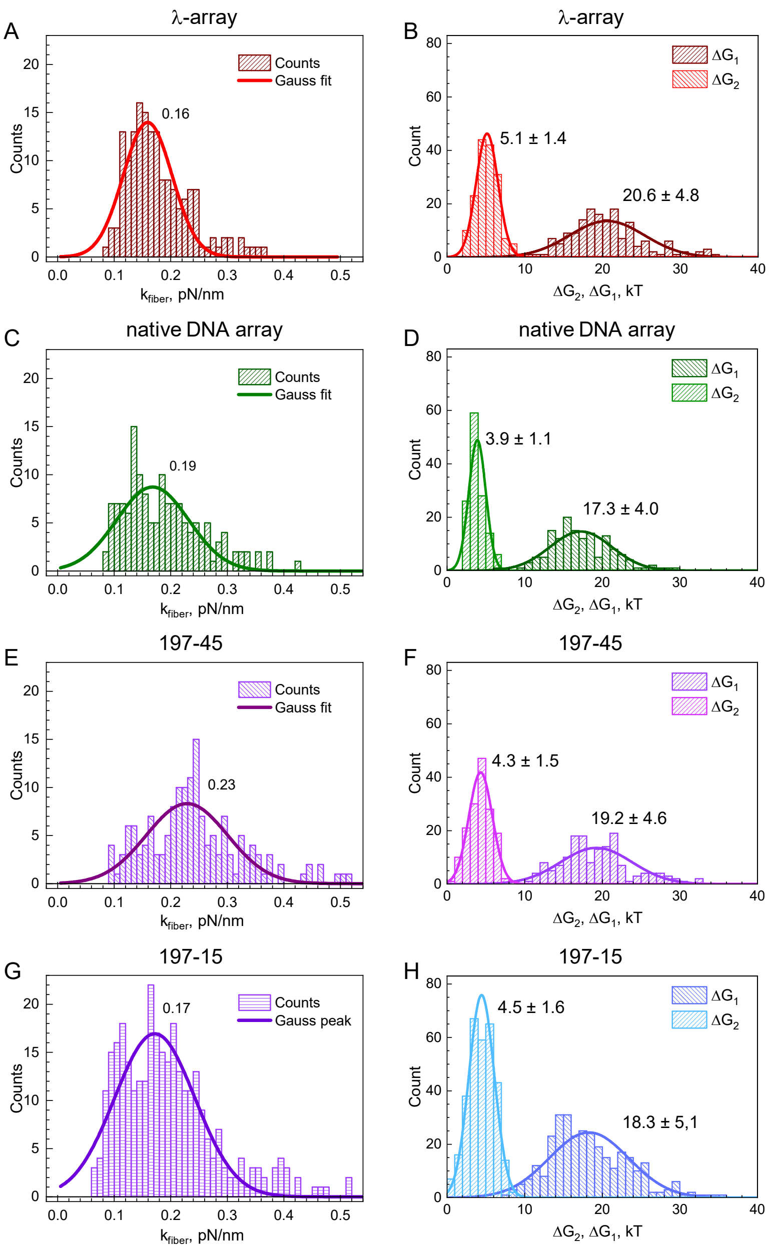

Figure S9. **Mechanical properties of the λ-arrays, arrays reconstituted on the MNase-digested genomic DNA, positioned 197 bp NRL arrays obtained for different HO:DNA ratios by fitting experimental data to the statistical mechanics model.** Since values of stiffness (k_{fiber}), energies of fiber unfolding (ΔG_1), and partial DNA unwrapping (ΔG_2) of the arrays do not show much dependence on the HO:DNA ratio (see **Fig. 5** of the main text), the data for each of these parameters were merged, and their distributions were fitted to the Gauss function. Lines are fittings to the single peak Gauss function with the maximum indicated in the panels. OriginPro software[11] (www.originlab.com) was used to create the graphs.

Supplementary Table S3

Table S3. Results of MMT studies of the nucleosome arrays reconstituted on the λ-DNA and DNAs with 197 bp nucleosome repeat length. Mean values of stiffness (k_{fiber}), energies of fiber unfolding (ΔG_1), and partial DNA unwrapping (ΔG_2) were calculated from all data of the respective dataset.

HO:DNA	Nucleosomes in array	Folded nucleosomes	k_{fiber} (pN/nm)	ΔG_1 ($k_B T$)	ΔG_2 ($k_B T$)	No of traces
**λ-arrays**; 48,548 bp DNA						
0.5	125 ± 60	49 ± 18	0.175 ± 0.052	20.0 ± 4.4	5.4 ± 1.6	57
0.8	187 ± 58	66 ± 26	0.182 ± 0.053	20.5 ± 5.8	4.5 ± 1.2	44
1.0	198 ± 69	68 ± 34	0.197 ± 0.086	22.4 ± 5.9	5.3 ± 1.4	89
pooled	--	--	0.18 ± 0.06	20.6 ± 4.8	5.1 ± 1.4	190
**native-DNA arrays**; 3,300 - 65,400 bp DNA						
0.5	7 - 155	5 - 84	0.190 ± 0.073	16.9 ± 3.6	3.9 ± 1.1	113
0.9 -1.0	18 - 286	10 - 110	0.188 ± 0.063	18.6 ± 4.5	4.0 ± 0.9	33
pooled	7 - 286	5 - 110	0.19 ± 0.08	17.3 ± 4.0	3.9 ± 1.1	146
**197-45 arrays**; 9,870 bp DNA						
0.5	27 ± 11	15 ± 8	0.238 ± 0.089	19.2 ± 4.7	4.3 ± 1.6	87
0.8	40 ± 13	20 ± 9	0.257 ± 0.097	19.9 ± 5.7	4.3 ± 1.4	63
1.0	35 ± 11	18 ± 5	0.237 ± 0.075	21.2 ± 6.3	4.9 ± 1.0	9
pooled	--	--	0.25 ± 0.09	19.2 ± 4.6	4.3 ± 1.5	159
**197-15 arrays**; 3,960 bp DNA						
0.5	14 ± 4	9 ± 4	0.185 ± 0.086	17.9 ± 5.0	4.4 ± 1.8	119
0.8	15 ± 4	9 ± 4	0.196 ± 0.087	17.8 ± 4.9	4.2 ± 1.6	104
1.0	17 ± 3	11 ± 4	0.208 ± 0.086	19.3 ± 5.4	4.8 ± 1.4	90
pooled	--	--	0.20 ± 0.09	18.3 ± 5.1	4.5 ± 1.6	313

Supplementary Table S4

Table S4. Results of the MMT studies of the nucleosome arrays reconstituted on the '601' positioning DNA sequences with various nucleosome repeat lengths. Mean values of stiffness (k_{fiber}), energies of fiber unfolding (ΔG_1), and partial DNA unwrapping (ΔG_2) are calculated from all data of the respective dataset. In the "Array" column, the first number indicates NRL; the second is for the number of nucleosome repeats in the DNA template.

Array	DNA length, bp**	Nucleosomes in array	Folded nucleosomes	k_{fiber} (pN/nm)	ΔG_1 (k_BT)	ΔG_2 (k_BT)	No of traces
177-36/38*	7,377/ 6,822	37 ± 9	21 ± 8	0.47 ± 0.24	17.1 ± 4.6	4.0 ± 1.3	428
172-25	5,305/ 4,396	24 ± 6	15 ± 5	0.41 ± 0.18	15.0 ± 3.8	4.3 ± 1.7	872
166-28	5,653/ 4,744	25 ± 6	16 ± 6	0.51 ± 0.28	13.9 ± 4.0	3.9 ± 1.5	480
162-27	5,379/ 4,470	26 ± 6	17 ± 6	0.53 ± 0.26	14.2 ± 3.9	4.2 ± 1.4	712

* Two DNA templates were studied, with 36 and 38 repeats: for the 177-36 DNA template, the total length of the DNA handles was 1,005 bp; 177-38 DNA had short handles of 102 bp total length.
** For the DNA templates, two numbers of the DNA length indicate two different preparations with long (1,005 bp) and short (102 bp) total lengths of the DNA handlers.

Supplementary references

1 Schalch, T. *The 30-nm chromatin fiber: in vitro reconstitution and structural analysis* PhD thesis, ETH, (2004).

2 Huynh, V. A. T., Robinson, P. J. J. & Rhodes, D. A method for the in vitro reconstitution of a defined “30 nm” chromatin fibre containing stoichiometric amounts of the linker histone. *J. Mol. Biol.* **345**, 957-968, doi:10.1016/j.jmb.2004.10.075 (2005).

3 Robinson, P. J. J., Fairall, L., Huynh, V. A. T. & Rhodes, D. EM measurements define the dimensions of the "30-nm" chromatin fiber: Evidence for a compact, interdigitated structure. *Proc. Natl. Acad. Sci. U.S.A.* **103**, 6506-6511, doi:10.1073/pnas.0601212103 (2006).

4 Kaczmarczyk, A., Brouwer, T. B., Pham, C., Dekker, N. H. & van Noort, J. Probing chromatin structure with magnetic tweezers. *Methods Mol. Biol.* **1814**, 297-323, doi:10.1007/978-1-4939-8591-3_18 (2018).

5 Brouwer, T. B., Kaczmarczyk, A., Pham, C. & van Noort, J. Unraveling DNA organization with single-molecule force spectroscopy using magnetic tweezers. *Methods Mol. Biol.* **1837**, 317-349, doi:10.1007/978-1-4939-8675-0_17 (2018).

6 Brouwer, T. B., Hermans, N. & van Noort, J. Multiplexed nanometric 3D tracking of microbeads using an FFT-phasor algorithm. *Biophys. J.* **118**, 2245–2257, doi:10.1016/j.bpj.2020.01.015 (2020).

7 Meng, H., Andresen, K. & van Noort, J. Quantitative analysis of single-molecule force spectroscopy on folded chromatin fibers. *Nucleic Acids Res.* **43**, 3578–3590, doi:10.1093/nar/gkv215 (2015).

8 Kaczmarczyk, A. *Nucleosome stacking in chromatin fibers probed with single-molecule force- and torque-spectroscopy* Ph. D. thesis, Leiden University, (2019).

9 Kaczmarczyk, A. *et al.* Single-molecule force spectroscopy on histone H4 tail cross-linked chromatin reveals fiber folding. *J. Biol. Chem.* **292**, 17506-17513, doi:10.1074/jbc.M117.791830 (2017).

10 Brouwer, T. *et al.* A critical role for linker DNA in higher-order folding of chromatin fibers. *Nucleic Acids Res.* **49**, 2537-2551, doi:10.1093/nar/gkab058 (2021).

11 OriginPro. Version 2019 (OriginLab Corporation, Northampton, MA, USA, 2019).
